# Prognostic Risk Signature and Comprehensive Analyses of Endoplasmic Reticulum Stress-Related Genes in Lung Adenocarcinoma

**DOI:** 10.1155/2022/6567916

**Published:** 2022-05-04

**Authors:** CaiZhen Yang, YuHui Wei, WenTao Li, JinMei Wei, GuoXing Chen, MingPeng Xu, GuangNan Liu

**Affiliations:** Department of Respiratory and Critical Care, The Second Affiliated Hospital of Guangxi Medical University, Nanning, China

## Abstract

Lung adenocarcinoma (LUAD) is the main pathological subtype of non-small-cell lung cancer. Endoplasmic reticulum stress (ERS) has been found to be involved in multiple tumor-related biological processes. At present, a comprehensive analysis of ERS-related genes in LUAD is still lacking. A total of 1034 samples from TCGA and GEO were used to screen differentially expressed genes. Further, Random Forest algorithm was utilized to screen characteristic genes related to prognosis. Then, LASSO Cox regression was used to construct a prognostic signature. Taking the median of signature score as the threshold, patients were separated into high-risk (HR) group and low-risk (LR) group. Tumor mutation burden (TMB), immune cell infiltration, cancer stem cell infiltration, expression of HLA, and immune checkpoints of the two risk groups were analyzed. TIDE score was used to evaluate the response of the two risk groups to immunotherapy. Finally, the gene expression was verified in clinical tissues with RT-qPCR. An eight-gene signature (ADRB2, AGER, CDKN3, GJB2, SFTPC, SLC2A1, SLC6A4, and SSR4) was constructed. TMB and cancer stem cell infiltration were higher in the HR group than the LR group. TIDE score and expression level of HLA were higher in the LR group than the HR group. Expression level of immune checkpoints, including CD28, CD27, IDO2, and others, were higher in the LR group. Multiple drugs approved by FAD, targeting ERS-related genes, were available for the treatment of LUAD. In summary, we established a stable prognostic model based on ERS-related genes to help the classification of LUAD patients and looked for new treatment strategies from aspects of immunity, tumor mutation, and tumor stem cell infiltration.

## 1. Introduction

Lung adenocarcinoma (LUAD) is the main subtype of non-small-cell lung cancer (NSCLC), accounting for 40% of all lung cancers [1]. The survival rate of LUAD is only 4% to 17% [2]. Molecular targeted therapy has made great progress in the treatment of LUAD, especially in advanced patients with specific gene aberrations, such as EGFR, KRAS, and BRAF [2]. However, most LUAD patients without specific gene aberrations did not benefit from it. Some immunotherapeutic drugs targeting PD-1 or PD-L1 have entered the clinic, and patients with LUAD were rapid and lasting response to them [3]. However, in addition to CTLA-4, PD-1 and its ligands, other immune checkpoints have not been further studied in the clinic, and a spectrum of toxicities of immunotherapy is inevitable [4]. To benefit more LUAD patients, it is essential to find new targeted genes and immune checkpoints.

Endoplasmic reticulum stress (ERS) can be caused by multiple factors, such as oxidative stress, nutritional deficiency, and oxygen limitation, leading to the production of misfolded proteins. When the accumulation of misfolded proteins exceeds the threshold, it will stimulate the unfolded protein response (UPR), which is a cell's corrective response. IRE1 *α* (inositol-requiring enzyme 1*α*), PERK (PKR-like ER kinase), and ATF6*α* (activating transcription factor 6*α*) are key endoplasmic reticulum transmembrane proteins that initiate UPR. There are two results after UPR work, restoring homeostasis or promoting cell death [5]. Previous research reported that protease inhibitors can induce ERS in myeloma cells and block the IRE1*α*/XBP-1 pathway, inducing apoptosis of myeloma cells [6]. The PERK-ATF4 pathway promoted the resistance of colon cancer cells to 5-fluorouracil, while the effect of PERK inhibitor was opposite [7]. ERS is related to many biological processes. Ferroptosis can induce ERS, and both ferroptosis and ERS can promote the expression of proapoptotic protein PUMA [8]. Hypoxia can cause ERS, which in turn increases the activity of hypoxia-related factors and hypoxia-related events [9]. Cells clean up the damaged endoplasmic reticulum caused by continuous ERS by autophagy [10]. Immunotherapy has made great progress in the treatment of LUAD, highlighting the importance of exploring the immune microenvironment [1]. Studies have found that endoplasmic reticulum stress is involved in immune-related biological processes, such as T-cell exhaustion and lymphocyte differentiation [11–13]. These studies show that ERS can directly affect tumor progression or indirectly affect tumor progression by participating in other important biological processes. However, there is no comprehensive study on the role of ERS-related genes in the biological characteristics and clinical prognosis of LUAD.

To understand the role of ERS in the prognosis and treatment of LUAD, we established a prognosis signature based on ERS-related genes and analyzed the signature in many aspects. Firstly, we downloaded the mRNA profile, mutation, and clinical data of LUAD from the public databases. Then, the roles of ERS-related genes in the prognosis, immunity, tumor mutation burden, and tumor cell stemness of LAUD were explored. Finally, multiple targeted drugs and immunotherapy drugs were identified for LUAD patients.

## 2. Methods

### 2.1. Material Source

The mRNA, mutation, and clinical data were downloaded from the TCGA database. The mRNA file contained 594 samples (535 LUAD samples and 59 nontumor samples). GSE40791, GSE31210, GSE30219, GSE41271, GSE50081, and GSE72094 from GEO were also included in this study. GSE40791 consisted of 94 LUAD tissues and 100 normal lung tissues. GSE31210 consisted of 226 LUAD tissues and 20 normal tissues. Samples in TCGA-LUAD, GSE40791, and GSE31210, a total of 1034, were used to conduct differential expression analysis. After removing samples with unknown follow-up time and survival status, there were 490 samples in TCGA-LUAD cohort for subsequent analyses. GSE41271, GSE30219, GSE50081, and GSE72094 were used as testing cohorts. In addition, a total of 785 ERS-related genes were obtained from the literature [14]. A total of 24 clinical tissues (12 LUAD tissues and 12 para-tumor tissues) were obtained in the Department of Respiratory and Critical Care, the Second Affiliated Hospital of Guangxi Medical University from September 2021 to January 2022. The acquisition of 24 clinical tissues of LUAD patients was approved by the Ethics Review Committee of the Second Affiliated Hospital of Guangxi Medical University (Approval Number: 2021-KY (0332)) and approved by patients with written informed consent.

### 2.2. Differentially Expressed Gene Screening

The differentially expressed ERS-related genes in TCGA-LUAD, GSE40791, and GSE31210 datasets were screened, respectively (∣logFC | >1 and FDR < 0.05). Then, the common differentially expressed genes in the three datasets were extracted for subsequent analysis. A heatmap was made using the “pheatmap” package to visualize common differentially expressed genes. The protein-protein interaction (PPI) network was performed in the STRING website (http://string.embl.de/) [15].

### 2.3. Functional Enrichment Analysis

To observe which pathways and functions that differentially expressed ERS-related genes were enriched, KEGG (Kyoto Encyclopedia of Genes and Genomes) and GO (Gene Ontology) were conducted using the “clusterProfiler” package. In order to understand whether ERS-related genes were involved in lung diseases, especially LUAD, DO (Disease Ontology) was also conducted using the R package ‘DOSE' [16].

### 2.4. Machine Learning and Prognostic Signature

Univariate Cox regression analysis was used to screen ERS-related genes that were related to prognosis. To achieve a high degree of prediction accuracy, Random Forest algorithm was further employed to screen important prognosis-related genes using the “randomForest” package. Genes with an importance score greater than 2 were used for subsequent signature establishment. The LASSO Cox regression analysis, which has the advantage of minimizing the risk of overfitting, was used to construct prognostic signature, using 10-fold cross-validation with the “glmnet” package. The formula was established as follows: Risk score = e^sum(eachgene'sexpression × correspondingcoefficient)^. Taking the median of risk score as the threshold, patients were separated into high-risk (HR) group and low-risk (LR) group. PCA and tSNE analyses were employed to observe whether the signature can clearly distinguish patients of two groups using the “Rtsne” package. To determine whether the signature was an independent predictor, univariate and multivariate Cox regression analyses were conducted.

### 2.5. Immune Microenvironment Analysis

Infiltration levels of stromal and immune cells can be calculated with the ESTIMATE algorithm [17]. ssGSEA (single-sample gene set enrichment analysis) can calculate an enrichment score, which indicates the enrichment degree of genes in a specific gene set. The process of ssGSEA includes ranking genes according to the absolute expression of genes in the sample and then calculating the enrichment score by integrating the differences between the empirical cumulative distribution functions of gene ranking [18, 19]. It was performed to calculate enrichment scores of sixteen immune cells using the “GSEABase” and “GSVA” packages. The correlation between six immune cell infiltration and gene expression was explored in the TIMER database. We also explored the effect of gene copy number variation on immune cell infiltration in the TIMER database. Six immune cells were B cell, macrophage, neutrophil, dendritic cell, CD8+ T cell, and CD4+ T cell.

To determine whether there were differences in immune checkpoint blockade (ICB) therapy between the HR and LR group, the expression of multiple immune checkpoint molecules was compared. The TCGA-LUAD cohort was used as training set and GSE72094 was used as testing set. Differentially expressed immune checkpoints between two groups were visualized. The TIDE (Tumor Immune Dysfunction and Exclusion) website (http://tide.dfci.harvard.edu/) provided TIDE scores on anti-PD-1 and anti-CTLA-4 responses in melanoma and NSCLC, providing the response of these two tumors to ICB therapy. TIDE score was negatively related to the response rate of ICB therapy [20].

### 2.6. Antigen Presentation Analysis

HLA (human leukocyte antigen), which is a protein encoded by MHC, is involved in the antigen presentation process. To observe whether there were differences in antigen presentation between the two groups, the expression level of HLA in the two risk groups was compared using the “limma” packages [21].

### 2.7. Tumor Mutation Burden and Gene Mutation Analysis

To observe whether there were genetic mutation differences between the HR and LR groups, tumor mutation burden (TMB) analysis was conducted. We combined the mutation level and risk score level to divide the patients into four groups and compared their survival time, including the high-TMB + HR group, low-TMB + HR group, high-TMB + LR group, and low-TMB + LR group. The landscape of genetic mutations was shown in a waterfall diagram. The mutation of signature genes was searched in the cBioPortal website (http://www.cbioportal.org).

### 2.8. Cancer Stem Cell Infiltration Analysis

Cancer stem cell, which is a small and rare subset of cells with stem cell-like characteristics, plays an important role in tumor proliferation, metastasis, and recurrence [22]. To observe the difference of stem cell infiltration between the two groups, we made a comparative analysis at the DNA and RNA levels. The independent variable was stress score, and the dependent variable was stem cell index, which represented the degree of stem cell infiltration.

### 2.9. Gene Set Enrichment Analyses

To observe whether there were differences in biological processes and pathways between the two groups, GSEA (gene set enrichment analysis) was utilized to conduct GO and KEGG analyses using the R package “org. Hs.eg. db”.

### 2.10. Drug Sensitivity Analysis

Based on grouping, we screened drugs for patients in HR or LR groups using the “pRRophetic” package [23]. IC50 as an evaluation index of drug sensitivity, the smaller it is, the more sensitive the patient is to the drug.

Drug sensitivity information file was obtained from the CellMiner (http://discover.nci.nih.gov/cellminer), which is a large database integrating different molecular types of NCI-60 and related metadata [24]. Then, targeting drugs were selected from the 263 FDA-approved drugs. The positive correlation indicated that the gene was sensitive to drugs, while the negative correlation was the opposite. The results of the first 16 analyses were visualized according to the *P* value from small to large row.

### 2.11. Verification of Signature Genes in Databases

The mRNA expression level of multiple LUAD cell lines was downloaded from the CCLE (Cancer Cell Line Encyclopedia, http://www.broadinstitute.org/ccle). The protein expression level was verified in the HPA (The Human Protein Atlas) database.

### 2.12. Verification of Signature Gene Expression with Clinical Samples

The mRNA of 24 clinical samples (12 LUAD tissues and 12 paratumor tissues) was extracted. Then, quantitative reverse transcription PCR (RT-qPCR) was used to verify the gene expression. TRIzol reagent was used to extract total RNA. cDNA synthesis kit and primers were purchased from Takara Biotechnology Co. (Dalian, China). GAPDH acted as the internal control for the RT-qPCR. The process of RT-qPCR was performed using the ABI7300 real-time fluorescence quantitative PCR instrument (Thermo Fisher Scientific, USA). The *Δ*Ct of paratumor tissues was normalized to calculate the *ΔΔ*Ct of LUAD tissues. The 2^−*ΔΔ*Ct^ method was used to calculate the relative gene expression [25].

### 2.13. Statistical Software

Except that the results of RT-qPCR were statistically analyzed using the SPSS 26.0 (IBM Corporation, Illinois, USA), other data were analyzed using the R.4.1.1 (R Core Team, Massachusetts, USA). Differentially expressed gene analysis, tumor mutation burden analysis, ssGSEA score, immune checkpoints analysis, and HLA analysis were conducted using the Mann–Whitney test. Cancer stem cell infiltration and drug sensitivity analyses were performed using Pearson's correlation test. The differences between clinical tissues were tested by Student's *t*-test. Log-rank test and the Kaplan-Meier analysis were used to compare the overall survival (OS) between groups.

## 3. Results

**Figure 1:**
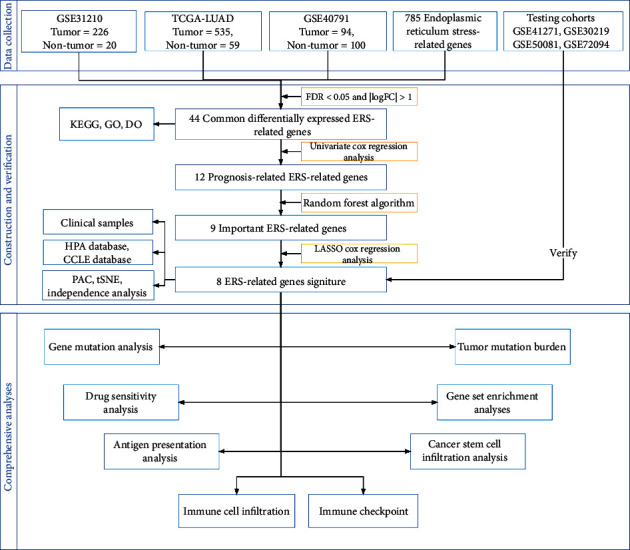
The research process of this study.

The flowchart of this study is shown in Figure 1. All 785 ERS-related genes are listed in Table S1.

### 3.1. Differentially Expressed Genes and Functional Enrichment Analysis

**Figure 2:**
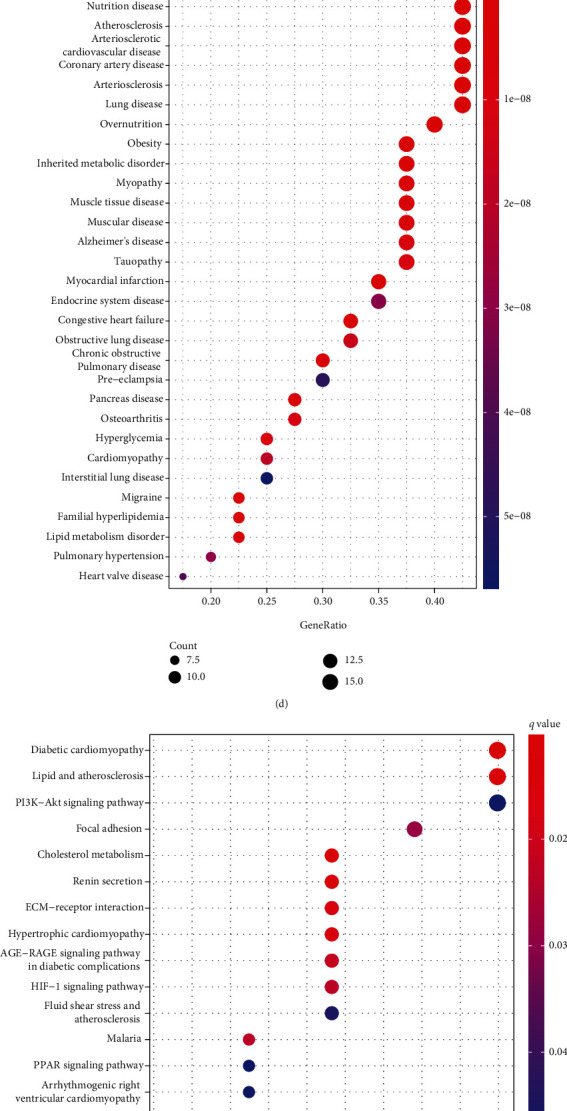
Total 44 common differentially expressed genes. (a) Venn Diagram. (b) Heatmap. (c) PPI of 44 genes. (d) DO analysis. (e) KEGG analysis. (f) GO analysis.

There were 44 common differentially expressed genes in TCGA-LUAD, GSE31210, and GSE40791, including 20 upregulated and 24 downregulated genes (Figures 2(a) and 2(b)). PPI showed that most genes were interrelated (Figure 2(c)). DO analysis demonstrated that they were associated with lung disease, lung adenocarcinoma, arteriosclerosis, and so on (Figure 2(d)). KEGG analysis showed that they were focused on focal adhesion, ECM-receptor interaction, PI3K-Akt signaling and HIF-1 signaling pathway, and so on (Figure 2(e)). GO analysis showed that these 44 genes were mainly enriched in cellular response to peptide, regulation of binding, lipid transport, and so on in the biological process; endoplasmic reticulum lumen, rough endoplasmic reticulum, cell-cell contact zone, and so on in the cellular component; peptide binding, amide binding, protein-lipid complex binding, and so on in the molecular functions (Figure 2(f)). As expected, differentially expressed genes were mainly involved in the biological processes and pathways of endoplasmic reticulum related functions and participate in the development of LUAD.

### 3.2. Machine Learning and Prognostic Signature

**Figure 3:**
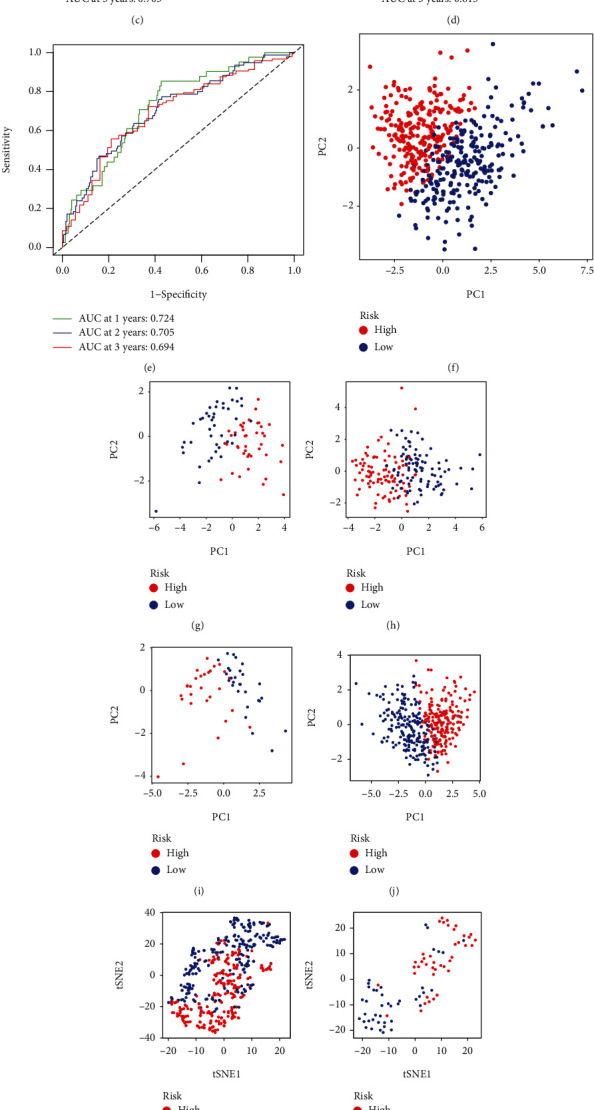
ROC, PCA, and tSNE analyses of training and testing sets. (a–e) ROC; (f–j) PCA analysis; (k–o) tSNE analysis. (a, f, and k) TCGA-LUAD. (b, g, and l) GSE30219. (c, h, and m) GSE41271. (d, i, and n) GSE50081. (e, l, and o) GSE72094.

Twelve genes were related to the prognosis of LUAD (Figure S1A). Further, nine characteristic genes were screened out by Random Forest algorithm (trees = 114) (Figure S1B, C). An eight-gene signature was constructed (Figure S1D, E). The calculation formula of the risk score was as follows: Risk score = (−0.204766423)∗ADRB2 + (−0.01476427)∗AGE*R* + 0.132930848∗CDKN3 + 0.08065608∗GJB2 + (−0.006115914)∗SFTPC + 0.071988516∗SLC2A1 + (−0.106235847)∗SLC6A4 + (−0.218749074)∗SSR4. Risk score of all testing cohorts was calculated according to the formula. The survival analysis of TCGA and validation cohorts showed that OS of patients in the HR group was significantly lower than in the LR group (Figure S2A-E). The AUC of TCGA cohort was 0.698 at 1 year, 0.666 at 2 year, and 0.682 at 3 year (Figure 3(a)). The AUC of GSE30219 at 1, 2, and 3 years was 0.837, 0.803, and 0.830 (Figure 3(b)). The AUC of GSE41271 at 1, 2, and 3 years was 0.630, 0.697, and 0.703 (Figure 3(c)). The AUC of GSE50081 at 1, 2, and 3 years was 0.814, 0.672, and 0.615 (Figure 3(d)). The AUC of GSE72094 at 1, 2, and 3 years was 0.724, 0.705, and 0.694 (Figure 3(e)). PCA and tSNE analysis showed that the signature can well distinguish between HR patients and LR patients (Figures 3(f)–3(o)). Also, scatterplots showing distribution of risk score were produced (Figure S3A-J). These indicated that the signature had robust power in predicting the prognosis of LUAD patients and distinguishing patients with different risk levels. The risk score was identified as an independent predictor of LUAD by two Cox regression analyses (Figure S3K and L).

### 3.3. Immunity Correlation Analysis

**Figure 4:**
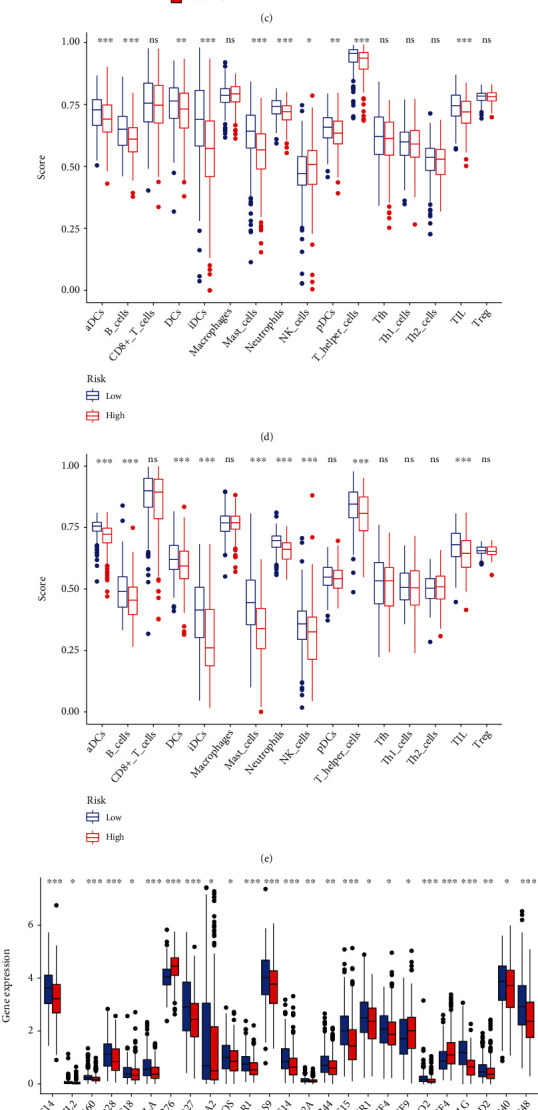
Immunity correlation analysis. (a) ESTIMATE score of immune cells. (b) ESTIMATE score of stromal cells. (c) TIDE score. (d) Abundance of immune cells in TCGA-LUAD cohort. (e) Infiltration score of immune cells in GSE72094. (f) Expression of immune checkpoints in TCGA-LUAD cohort. (g) Expression of immune checkpoints in GSE72094.

The ESTIMATE score of immune cells was positively correlated with the risk score, while the relationship between ESTIMATE score of stromal cells and the risk score was not statistical significance (Figures 4(a) and 4(b)). Next, we compared the immune cell scores of two groups. Both training and testing cohorts showed that the infiltration score of most immune cells in the LR group was higher than that in the HR group, such as aDCs, B cells, and T helper cells (Figures 4(d) and 4(e)).

The expressions of multiple immune checkpoints in the HR group, such as CD28, CD27, and IDO2, were significantly higher than that in the LR group. However, there was no difference in the expression of PD-1/PD-L1/PD-L2 and CTLA-4 between two groups (Figures 4(f) and 4(g)). TIDE score of the LR group was higher than the HR group, indicating that the LR group was more prone to immune escape than the HR group, and the effectiveness of immunotherapy of the HR group was better (Figure 4(c)).

**Figure 5:**
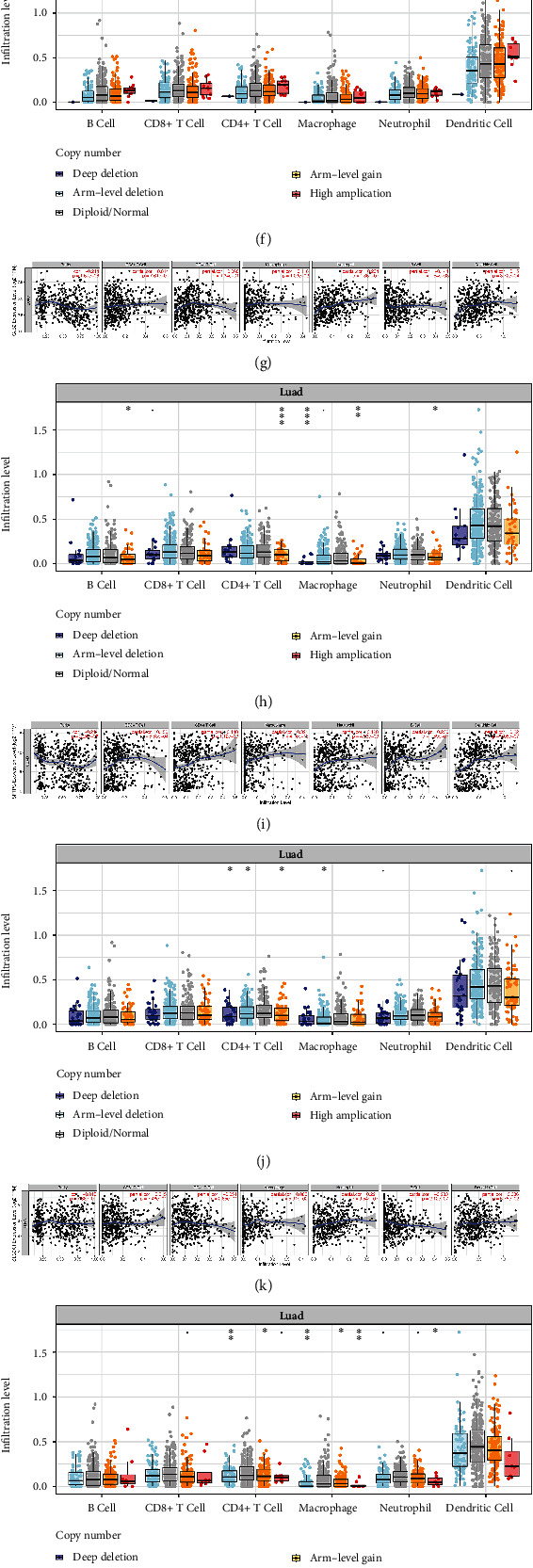
Eight genes and immune cell infiltration. (a and b) ADRB2. (c and d) AGER. (e and f) CDKN3. (g and h) GJB2. (i and j) SFTPC. (k and l) SLC2A1. (m and n) SLC6A4. (o and p) SSR4.

It was found that the infiltration of immune cells was related to eight signature genes. CDKN3 was negatively correlated with CD4+ T cell, macrophage, B cell, and dendritic cell and positively correlated with CD8+ T cell and neutrophil (Figure 5(e)). SLC2A1 was negatively related to CD4+ T cell and B cell and positively related to other four immune cells (Figure 5(k)). GJB2 was negatively correlated with B cell and positively correlated with other five immune cells (Figure 5(g)). SSR4 was positively correlated with B cell and negatively correlated with other five immune cells (Figure 5(o)). ADRB2, AGER, SFTPC, and SLC6A4 were positively correlated with all six immune cells (Figures 5(a), 5(c), 5(i), and 5(m)).

Compared with the diploid/normal group, copy number variation of genes could affect the immune cell infiltration. Arm-level deletion of ADRB2 and CDKN3 decreased the abundance of six immune cells (Figures 5(b) and 5(f)). Arm-level gain of AGER decreased the infiltration of six immune cells. GJB2 and SFTPC were lack of high amplification (Figures 5(h) and 5(j)). Arm-level gain of GJB2 was negatively associated with infiltration of B cell, CD4+ T cell, macrophage, and neutrophil. Deep deletion, arm-level deletion, and arm-level gain of SFTPC were negatively related to the infiltration of CD4+ T cell. Copy number variation of SLC2A1 mainly affected CD4+ T cell and macrophage (Figure 5(l)). SLC6A4 and SSR4 did not have deep deletion (Figures 5(n) and 5(p)). Arm-level deletion of SLC6A4 negatively affected the infiltration of CD4+ T cell and macrophage. Arm-level deletion of SSR4 decreased the infiltration of macrophage, neutrophil, and dendritic cell.

### 3.4. Antigen Presentation Analysis

**Figure 6:**
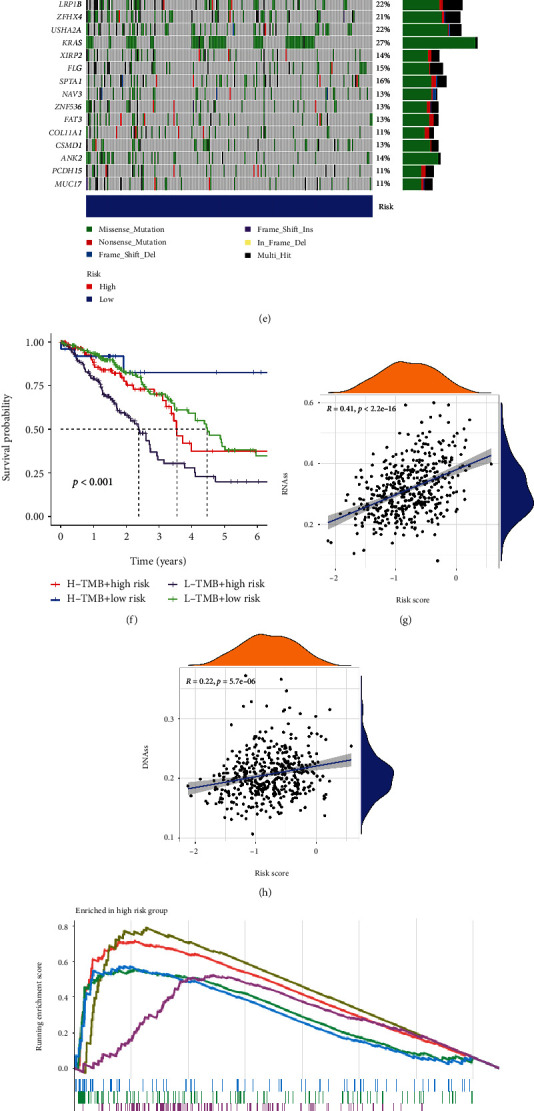
TMB, GSEA, and expression of HLA. (a) Expression of HLA in TCGA-LUAD cohort. (b) Expression of HLA in GSE72094. (c) TMB. (d) The landscape of genetic mutations of HR group. (e) The landscape of genetic mutations of LR group. (f) Survival analysis of risk score combined with TMB. (g) Evaluation of infiltration of cancer stem cells at RNA level. (h) Evaluation of infiltration level of cancer stem cells at DNA level. (i) KEGG of the HR group. (j) KEGG of the LR group. (k) GO of the HR group. (l) GO of the LR group.

There was significant difference in the expression of HLA related to antigen presentation between the HR group and LR group. In both training and test cohorts, HLA class I (including HLA-E and HLA-F) was higher in the LR group than in the HR group. The expression of multiple HLA class II, such as HLA-DMA, HLA-DMB, and HLA-DOA, was also higher in the LR group (Figures 6(a) and 6(b)).

### 3.5. Tumor Mutation Burden and Gene Mutation Analysis

TMB of the HR group was higher than the LR group (Figure 6(c)). The mutation frequency of most genes in the HR group was higher, such as TP53, TTN, MUC16, and RYR2 (Figures 6(d) and 6(e)). The OS of high-TMB + HR group was higher than of low-TMB + HR group, and the OS of high-TMB + LR group was better than of low-TMB + LR group (Figure 6(f)). Eight signature genes had differences in the mutation frequency and types (Figure S4A-H). Among the eight genes, SFTPC had the highest mutation frequency, which was 5%, and SLC2A1 and CDKN3 had the lowest mutation frequency, which was 1.2% (Figure S4I).

### 3.6. Cancer Stem Cell Infiltration Analysis

The stem cell infiltration analysis exhibited that the higher the risk score, the higher the degree of stem cell infiltration. The value of *R* was 0.2 (*P* = 2.1e − 05) in the DNA score, and 0.39 (*P* < 2.2e − 16) in the RNA score **(**Figures 6(g) and 6(h)**).** It indicated that LUAD cells in the HR group had more obvious stem cell characteristics and lower degree of cell differentiation.

### 3.7. Gene Set Enrichment Analyses

KEGG showed that pathways enriched in the HR group include cell cycle, DNA replication, and P53 signaling pathway, and pathways enriched in the LR group include asthma, autoimmune thyroid disease, and alpha linolenic acid metabolism (Figures 6(i) and 6(j)). In addition, GO showed that genes of the HR group were enriched in cell cycle checkpoint, cell cycle G2 phase transition, centromere complex assembly, chromatin assembly or disassembly, and so on, and genes of the LR group were involved in B cell-mediated immunity, axoneme assembly, antigen receptor mediated-signaling pathway, activation of immune response, and so on (Figures 6(k) and 6(l)).

### 3.8. Drug Sensitivity Analysis

**Figure 7:**
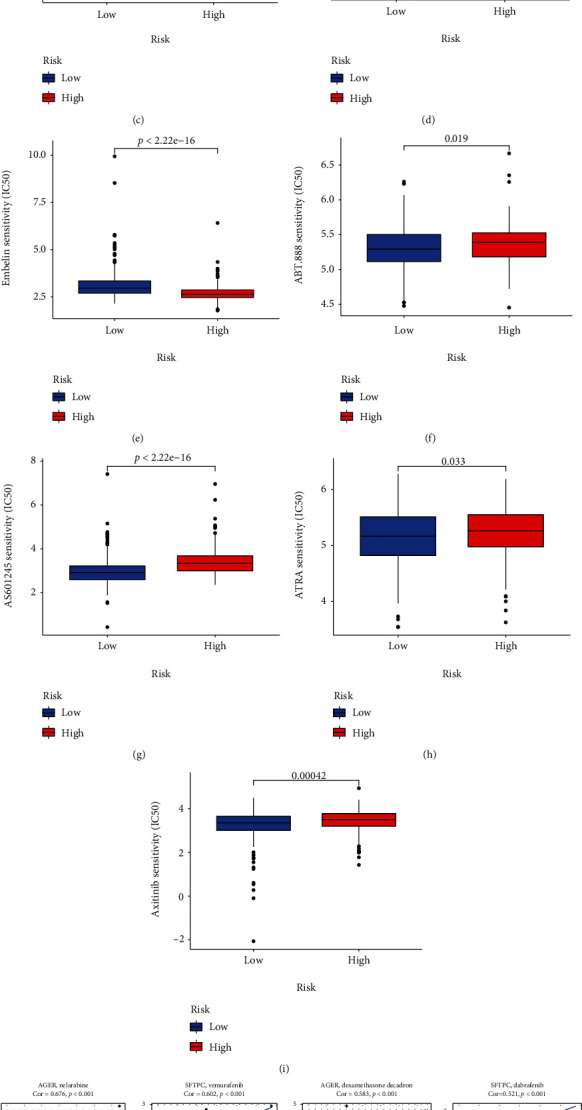
Drug sensitivity analysis. (a–i) Drugs for two groups. (a) Docetaxel. (b) Erlotinib. (c) AG.014699. (d) AKT.inhibitor.VIII. (e) Embelin. (f) ABT.888. (g) AS601245. (h) ATRA. (i) Axitinib. (j) Multiple drugs for eight signature genes.

The IC50 of docetaxel, erlotinib, AG.014699 (also known as rucaparib), AKT.inhibitor.VIII, and embelin were lower in the HR group than the LR group, suggesting that patients in the HR group were more sensitive to these drugs (Figures 7(a)–7(d)). The IC50 of ABT.888 (also known as veliparib), AS601245, ATRA, and axitinib were higher in the HR group than the LR group (Figures 7(e)–7(i)).

To provide reference for gene targeted therapy, we investigated the relationship between eight signature genes and drug efficacy in NCI-60 cell lines, which included LUAD cell lines. The results of the first 16 analyses were visualized according to the *P* value from small to large row (Figure 7(j)). AGER, SLC6A4, and SSR4 were all sensitive to fluphenazine, AGER and GJB2 were sensitive to gemcitabine, ADRB2 was sensitive to dacomitinib. SFTPC was sensitive to Denileukin Diftitox Ontak, while CDKN3 and SLC2A1 were resistant to it. The names of genes and drugs and their correlation coefficients were also provided in Table S2.

### 3.9. Verification of Signature Genes

**Figure 8:**
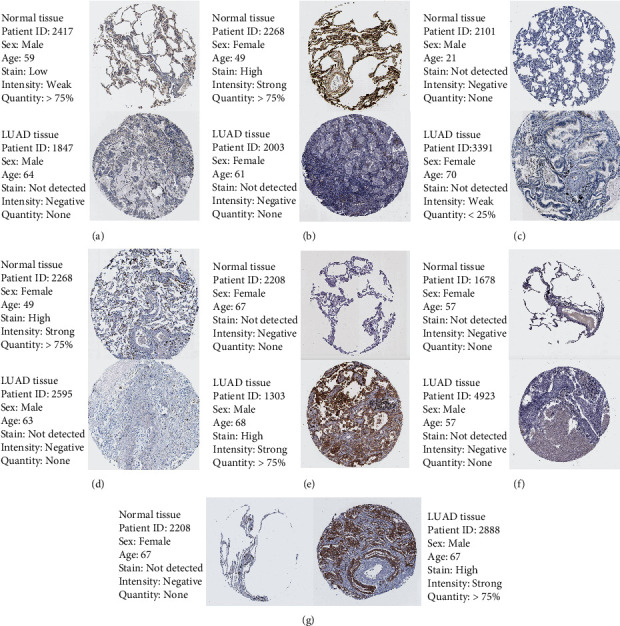
HPA database. (a) ADRB2. (b) AGER. (c) GJB2. (d) SFTPC. (e) SLC2A1. (f) SLC6A4. (g) SSR4.

**Figure 9:**
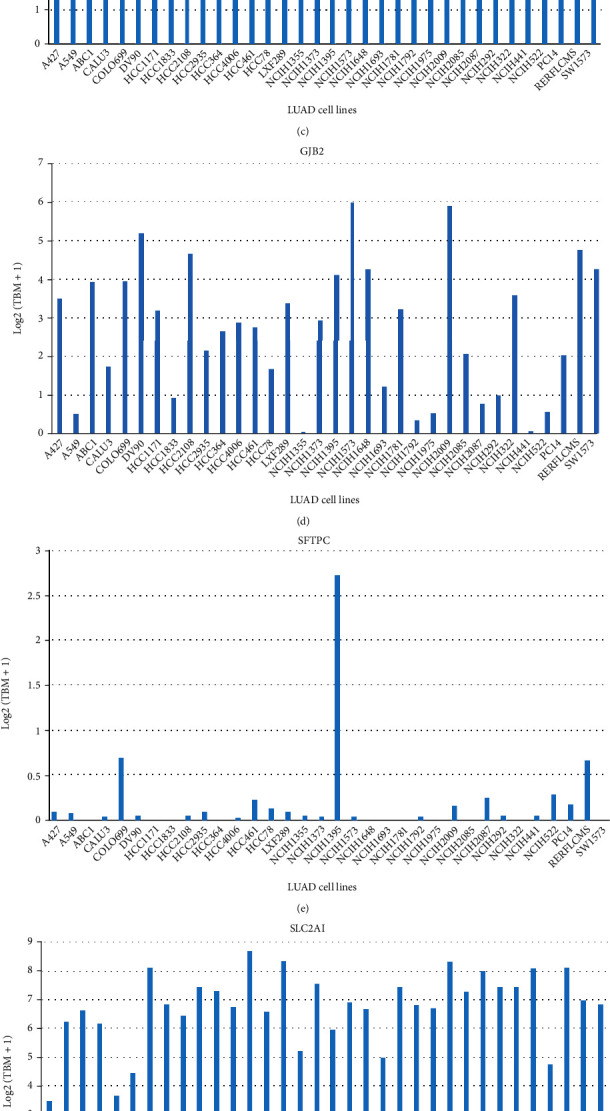
Gene expression in different LUAD cell lines. (a) ADRB2. (b) AGER. (c) CDKN3. (d) GJB2. (e) SFTPC. (f) SLC2A1. (g) SLC6A4. (h) SSR4.

**Figure 10:**
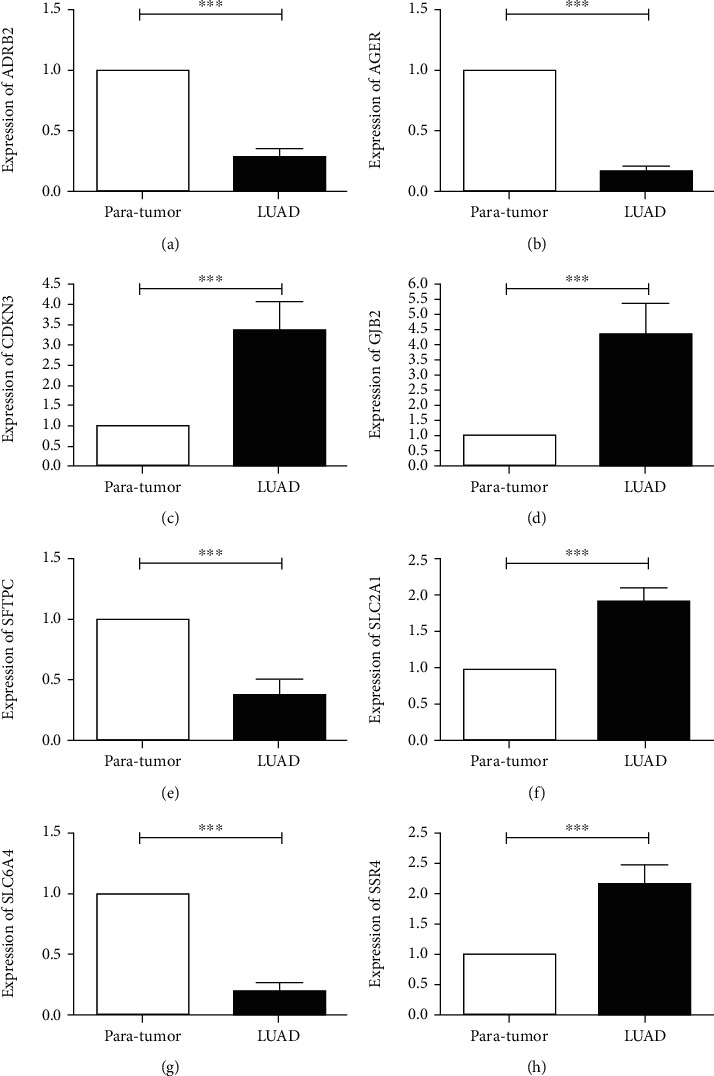
Verification of gene expression with clinical tissues. (a) ADRB2. (b) AGER. (c) CDKN3. (d) GJB2. (e) SFTPC. (f) SLC2A1. (g) SLC6A4. (h) SSR4. ^∗^ *P* < 0.05, ^∗∗^ *P* < 0.01, ^∗∗∗^ *P* < 0.001.

We further verified the expression of seven signature genes in the HPA database, founding the SLC2A1, SSR4, and GJB2 were upregulated, and AGER, SFTPC, SLC6A4, and ADRB2 were downregulated (Figures 8(a)–8(g)). The expression of the same gene was different in different LUAD cell lines (Figures 9(a)–9(h)). Between LUAD tissues and its counterpart, there was significant difference in expression of signature genes (Figures 10(a)–10(h)). The primer sequences are provided in Table S3 and the clinical information of all patients is provided in Table S4.

## 4. Discussion

LUAD is the main subtype of NSCLC [26]. In recent years, researchers have studied the pathogenesis of LUAD from different pathways and biological processes and made great progress in LUAD targeted therapy and immunotherapy. However, greater efforts are still needed. ERS is involved in many biological processes, such as ferroptosis [8], hypoxia [9], and autophagy [10]. So, it is worth exploring the role of ERS in LUAD.

To build a signature with stable prediction performance, we used a large number of samples (1034 in total) to screen the differentially expressed ERS-related genes. Then, Random Forest tree algorithm, which uses bootstrap aggregation and randomization of predictors based on importance filtering [27], was used to identify important genes. Eventually, LASSO, which is also a kind of machine learning and a popular method for regression with high-dimensional predictors [28], was used to construct an eight-gene prognostic signature, including ADRB2, AGER, CDKN3, GJB2, SFTPC, SLC2A1, SLC6A4, and SSR4. ADRB2/PKA pathway inhibited the apoptosis of prostate cancer cells and propranolol targeting ADRB2 can reduce the mortality of prostate cancer [29]. AGER, which is a member of the immunoglobulin superfamily, was detected at low expression level in NSCLC tissue and inhibited the proliferation of NSCLC cells [30]. However, it was upregulated in cervical cancer, promoting proliferation and migration of cervical cancer cells [31]. Mir-181d-5p suppressed the biological behavior of NSCLC cells by inhibiting CDKN3/Akt signaling pathway [32]. GJB2, which was a downstream target of CAR10, was upregulated in NSCLC and induced NSCLC cell migration [33]. The low expression of SFTPC could lead to poor prognosis in LUAD patients, and mir-629-3p could enhance the low expression of SFTPC [34]. The lncRNA PVT1/mir-378c/SLC2A1 axis was involved in the regulation of LUAD proliferation [35]. SLC6A4, which is a stress-related gene, could regulate transcription through epigenetic mechanism [36]. Compared with young B-cell chronic lymphoblastic leukemia (B-CLL) cells, aged B-CLL cells downregulated the expression of SLC6A4 [37]. SSR4 was a subunit of TRAP (translocation associated protein), and its deletion would cause congenital disorders of glycosylation [38]. At present, there are few studies on SSR4 in cancers. It showed that eight genes affected the infiltration of immune cells, which may suggest that these genes can affect the biological process of tumor by affecting the infiltration of immune cells. Gene copy number variation would affect the degree of immune cell infiltration. The AUC of all four testing sets was high, demonstrating the prediction accuracy and stability of the signature. We also screened drugs targeting these genes, such as gemcitabine, which is effective for NSCLC whether used in combination with other drugs or alone [39]. And Daktinib, which is an irreversible epidermal growth factor receptor (EGFR) tyrosine kinase inhibitor (TKI), significantly improved survival in patients with EGFR mutation positive NSCLC [40].

In our study, immune cell infiltration analysis showed that infiltration of antigen-presenting cells, such as DCs, neutrophils, and B cells, was higher in the LR group. DC cells can be divided into conventional type 1 (cDC1s), conventional type 2 (cDC2s), and plasmacytoid DC (pDCs) [41, 42]. In the mouse model, loss of cDC1s in the lung increased the tumor burden and decreased the infiltration of CD8 + T cells [43]. cDC2s played an antitumor role by presenting tumor-derived antigen to CD4+ conventional T cells [44]. pDCs promoted the production of Tregs, which can inhibit antitumor immunity and facilitate the immune escape of tumor cells [45]. Previous studies have opposite views on the role of neutrophils in tumors, but so far, the literature on the role of neutrophils in promoting tumor growth had greatly exceeded the literature on the role of neutrophils in inhibiting tumor growth [46]. Subpopulations of B cells play an opposite role in antitumor immunity. Bregs inhibited antitumor immunity by inhibiting antitumor cells [47], while TIL-B cells were involve in the process of anti-NSCLC [48]. Also, the infiltration level of TILs, which have antitumor effects and have become the focus of immunotherapy [49], was higher in the LR group. HLA-I and HLA-II are mainly expressed by antigen-presenting cells [50]. So accordingly, the expression of HLA-I and HLA-II was consistent with the infiltration of antigen-presenting cells, which was higher in the LR group. It was reported that the induction of ERS and the activation of UPR can inhibit the expression of MHC-I molecules [51]. When MHC-I was reduced or eliminated, tumor cells would escape immune supervision [50]. MHC-II was also expressed by tumor cells, such as NSCLC cells [52]. These results may explain why the OS of the LR group was higher than that of the HR group.

In a part of NSCLC patients, the use of antibodies targeting PD-1 and CTLA-4 had shown significant efficacy in clinical practice [53–55]. However, this mainly benefits patients who express higher PD-1 and CTLA-4. In this study, we found no difference in the expression of PD-1 or CTLA-4 between the HR group and LR group. However, we found that other immune checkpoints were differentially expressed between the two groups, such as CD28, CD27, and IDO2. The drugs for these immune checkpoints can be used alone or in combination with other immunotherapeutic drugs and have the advantage of low toxicity. For instance, tumor targeting CD28 bispecific antibody, which had small side effects, could enhance the antitumor efficacy of PD-1 immunotherapy, prolonging antitumor immunity [56]. Targeting CD27 antibody synergizes with PD-L1 blockade to enhance the activation of CD4+ T cells and the proliferation and function of CD8+ T cells [57]. The silencing of IDO2 in DC not only negatively regulated the growth of tumor cells but also helped to improve the immunotherapeutic effect of DC-based cancers [58]. Interestingly, although there was no difference in PD-1 and CTLA-4 expression between the HR and LR groups, TIDE score was high in the HR group. Further, we also found a variety of drugs that patients in the HR group were more sensitive to, such as erlotinib, docetaxel, and gefitinib. Some drugs that patients in the LR group were more sensitive to include methotrexate, ABT.888, and axitinib.

TMB, which represents the number of tumor-derived new antigens, is a key determinant of ICB response, and higher somatic TMB is related to better OS [59]. In this study, the OS of high-TMB + HR group was better than of low-TMB + HR group, and the OS of high-TMB + LR group was better than of low-TMB + LR group, suggesting that higher TMB is beneficial to OS of LUAD patients. Cancer stem cells have a strong ability to renew and replicate the heterogeneity of primary tumors [60]. The infiltration of cancer stem cells in the HR group was higher than the LR group, indicating that LUAD cells in HR group had more obvious stem cell characteristics and lower degree of cell differentiation. These may also explain why the lower OS in the HR group.

Multiple analyses were conducted, and experimental verification was carried out. However, there were still some deficiencies in this study. Only gene mRNA expression had been completely verified. The verification of CDKN3 protein was lacking because there was no CDKN3 expression data in HPA database. At the same time, there was a lack of functional experiments in vitro and in vivo to verify the effect of gene expression on the biological process of LUAD. What's more, it was unclear why the expression of PD-1 and CTLA-4 did not differ significantly between the two groups, while the difference of TIDE score between the two groups was statistically significant. It is necessary to carry out corresponding experiments in the future.

## 5. Conclusion

A stable prognostic signature was established based on ERS-related genes to help the classification of LUAD patients. We also comprehensively analyzed and looked for new treatment strategies from aspects of immunity, TMB, and tumor stem cell infiltration. What's more, multiple drugs for targeting genes and different groups of patients were screened.

## Data Availability

Data cohorts in this study are available from TCGA-LUAD, GSE31210, GSE40791, GSE30219, GSE41271, GSE50081, and GSE72094.

## Abbreviations

LUAD: Lung adenocarcinoma
ERS: Endoplasmic reticulum stress
HR: High risk
LR: Low risk
TMB: Tumor mutation burden
HLA: Human leukocyte antigen
TIDE: Tumor immune dysfunction and exclusion
NSCLC: Non-small-cell lung cancer
UPR: Unfolded protein response
KEGG: Kyoto Encyclopedia of Genes and Genomes
GO: Gene Ontology
DO: Disease Ontology
ICB: Immune checkpoint blockade
GSEA: Gene set enrichment analysis
CCLE: Cancer Cell Line Encyclopedia
RT-qPCR: Quantitative reverse transcription PCR.

## Supplementary Materials

Supplementary 1 Figure S1: (A) Univariate Cox regression for prognosis-related genes. (B) Random Forest algorithm. Find the point with the smallest cross-validation error and find the number of trees corresponding to this point. (C) Random Forest algorithm. Importance score of twelve prognosis-related genes. (D, E) LASSO Cox regression analysis.

Supplementary 2 Figure S2: survival analysis. (A) TCGA-LUAD. (B) GSE30219. (C) GSE41271. (D) GSE50081. (E) GSE72094.

Supplementary 3 Figure S3: independence analysis, distribution of risk score, and scatterplots of the correlation between risk scores and survival time.

Supplementary 4 Figure S4: gene mutation. (A) ADRB2. (B) AGER. (C) CDKN3. (D) GJB2. (E) SFTPC. (F) SLC2A1. (G) SLC6A4. (H) SSR4. (I) Mutation summary of eight genes.

Supplementary 5 Table S1: ERS-related genes obtained from the previous literature (total 785).

Supplementary 6 Table S2: drugs.

Supplementary 7 Table S3: gene primer sequence.

Supplementary 8 Table S4: clinical information of LUAD patients.
