# Supplementary figures and images for: Prognostic Risk Signature and Comprehensive Analyses of Endoplasmic Reticulum Stress-Related Genes in Lung Adenocarcinoma
Source: J Immunol Res. 2022 May 4;2022:6567916. doi: 10.1155/2022/6567916 (PMC9096573; doi:10.1155/2022/6567916)

A

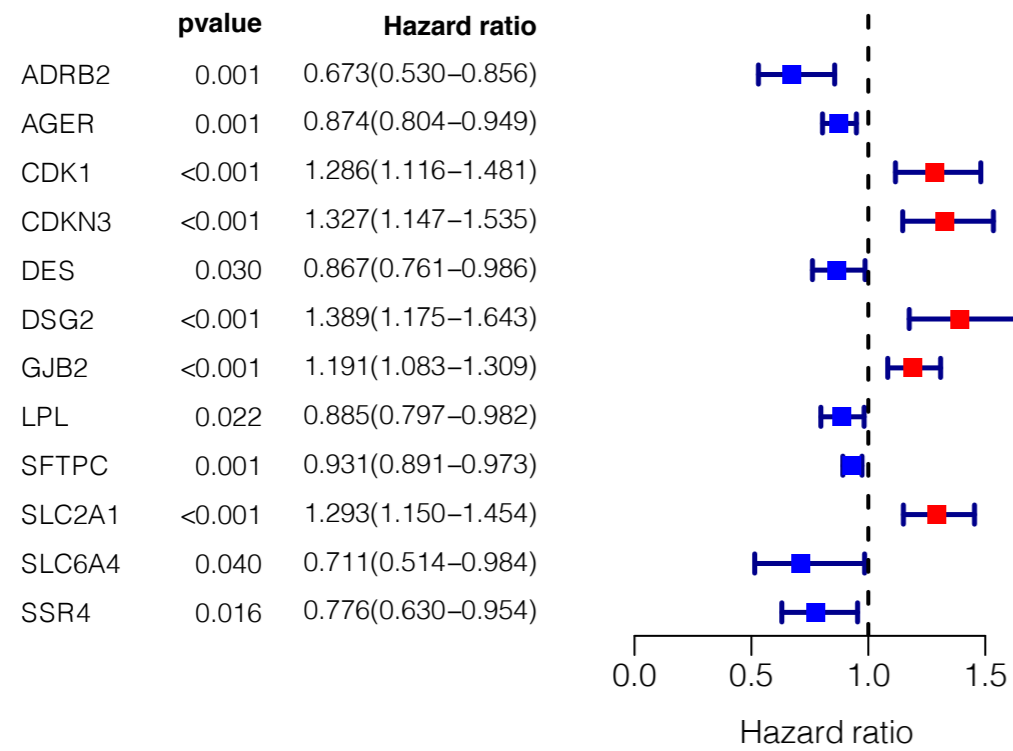

B

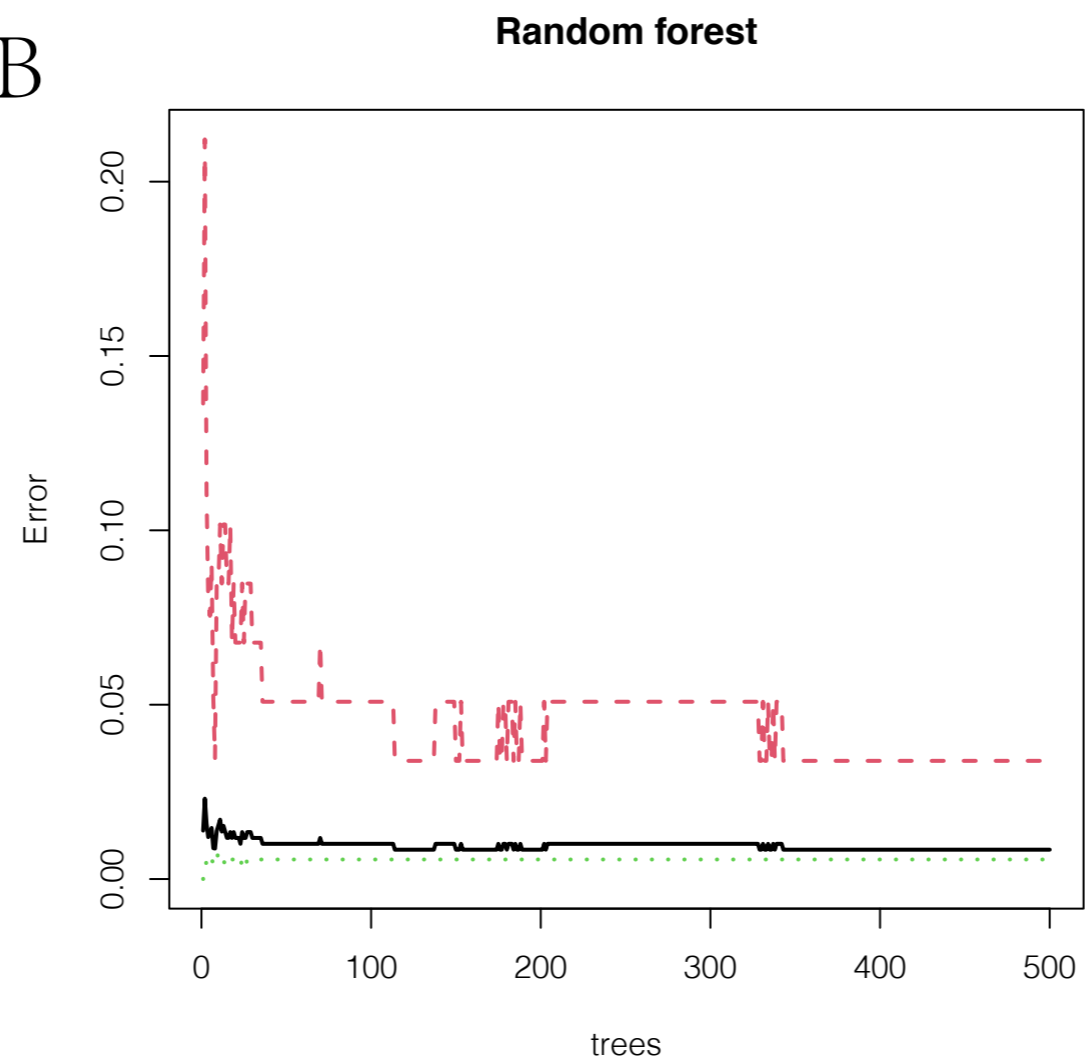

C

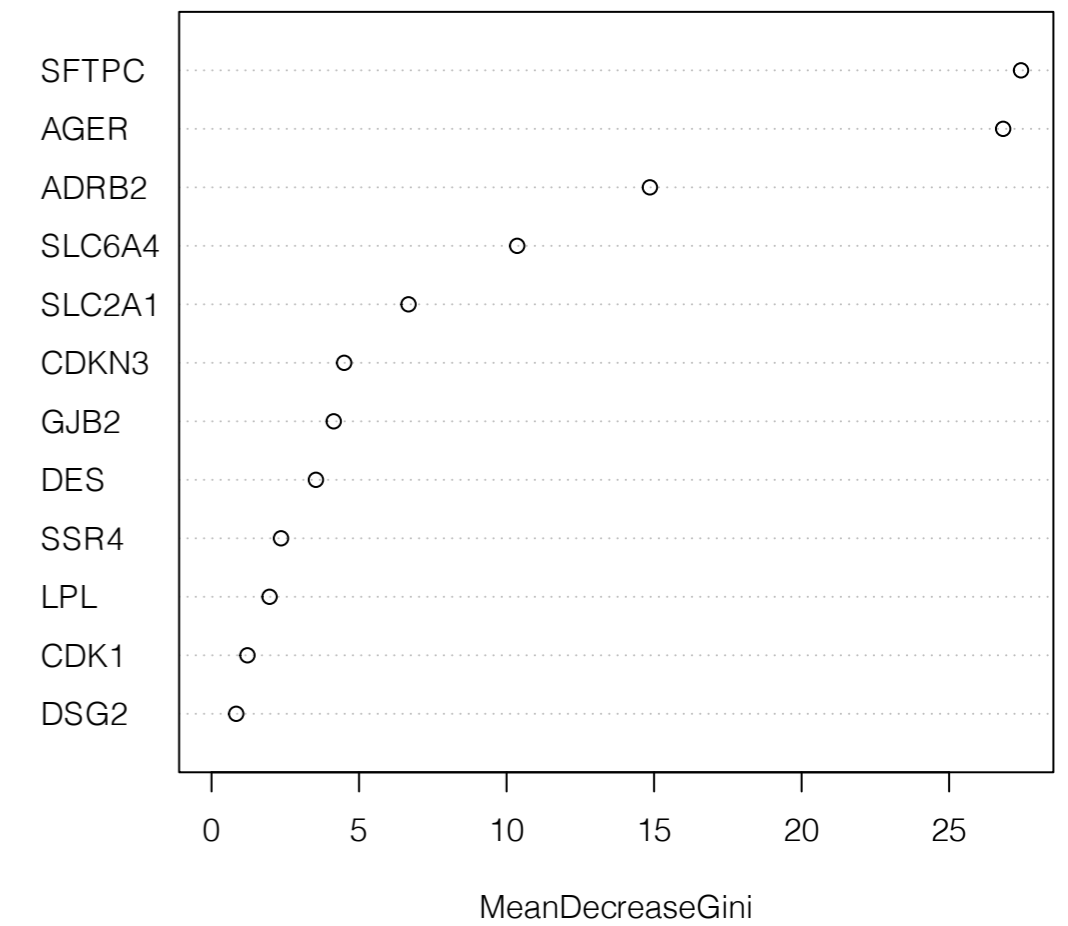

D

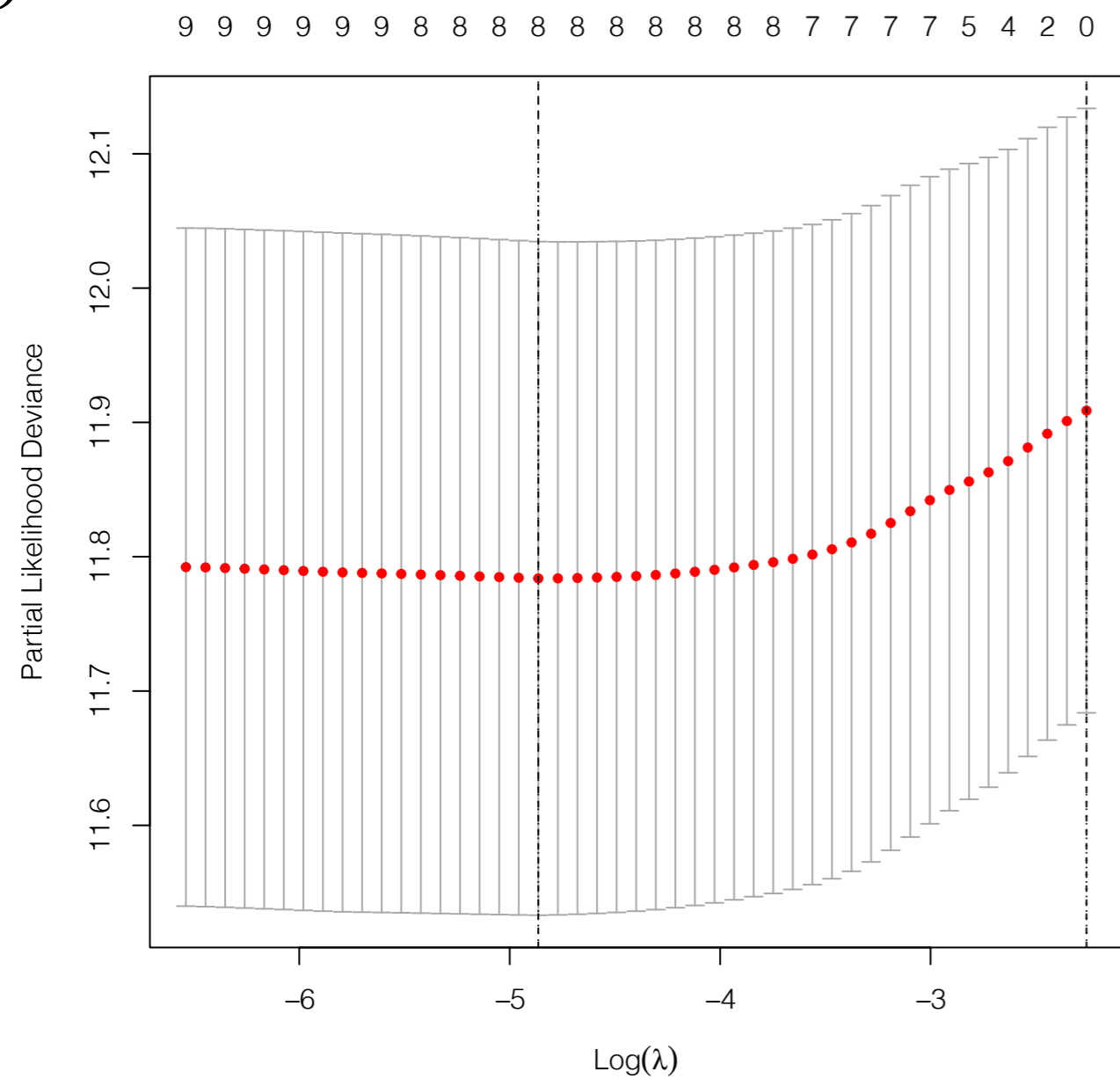

E

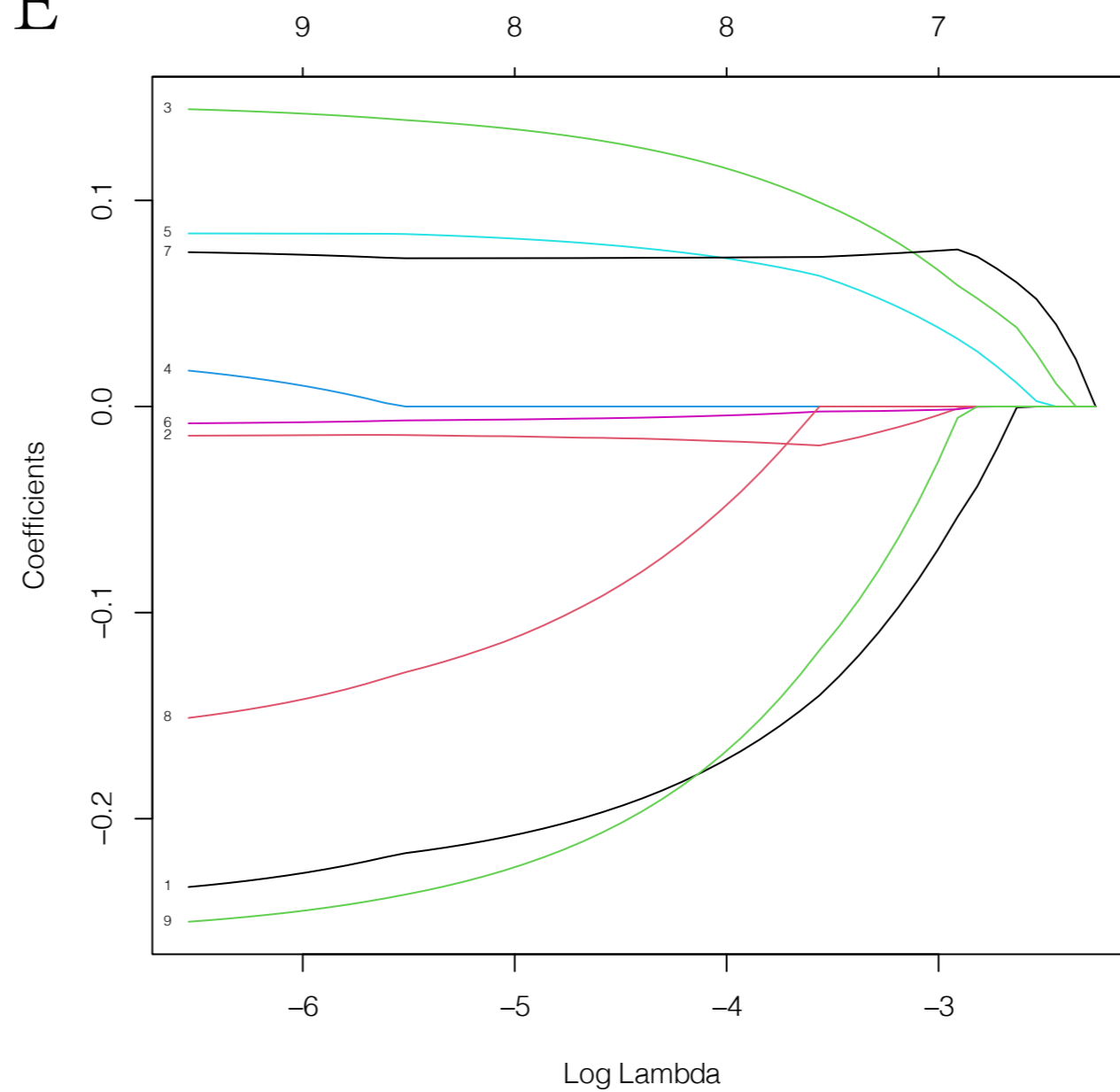

Supplement: Supplementary 1 — Figure S1: (A) Univariate Cox regression for prognosis-related genes. (B) Random Forest algorithm. Find the point with the smallest cross-validation error and find the number of trees corresponding to this point. (C) Random Forest algorithm. Importance score of twelve prognosis-related genes. (D, E) LASSO Cox regression analysis. [file 6567916.f1.pdf]

A

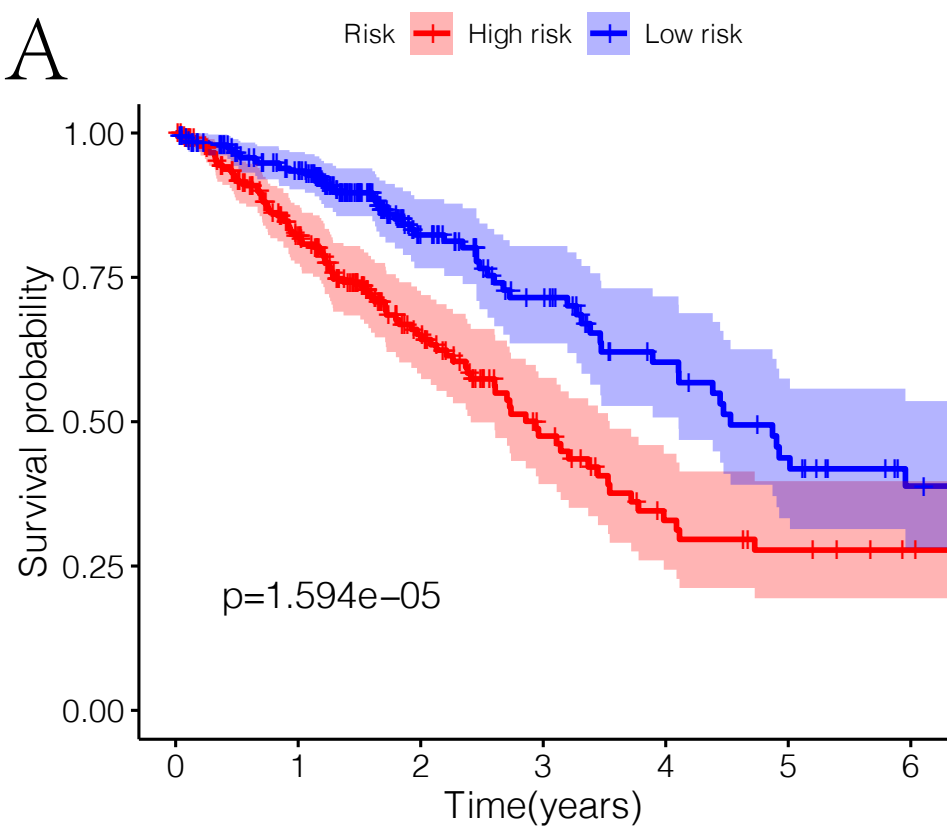

B

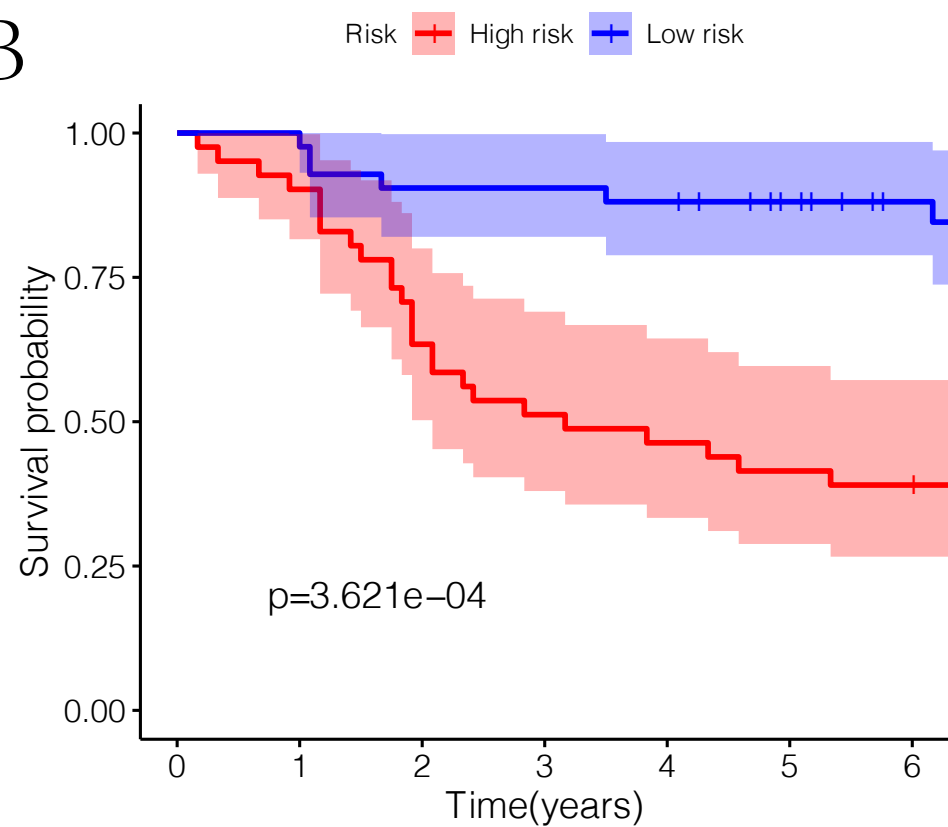

C

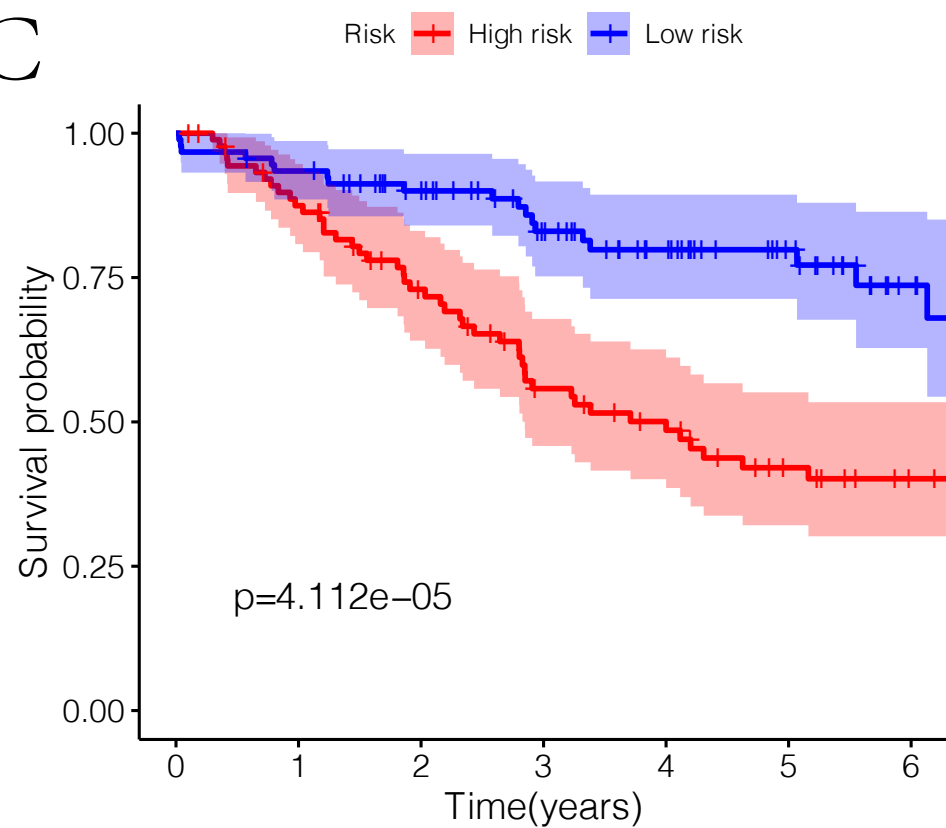

D

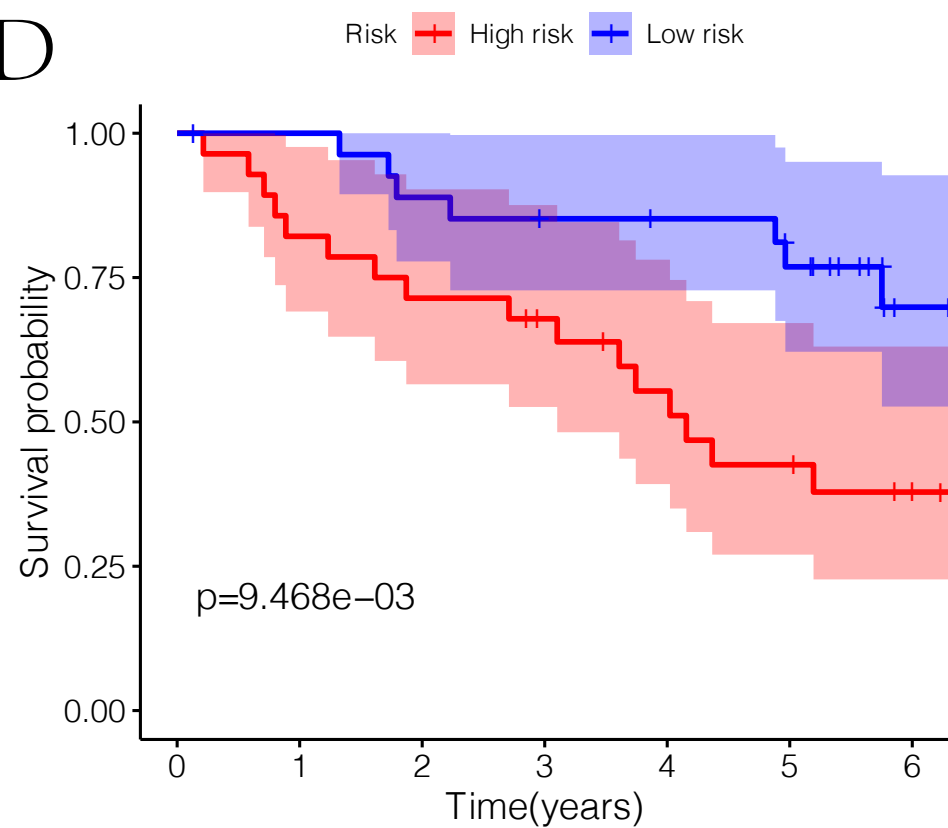

E

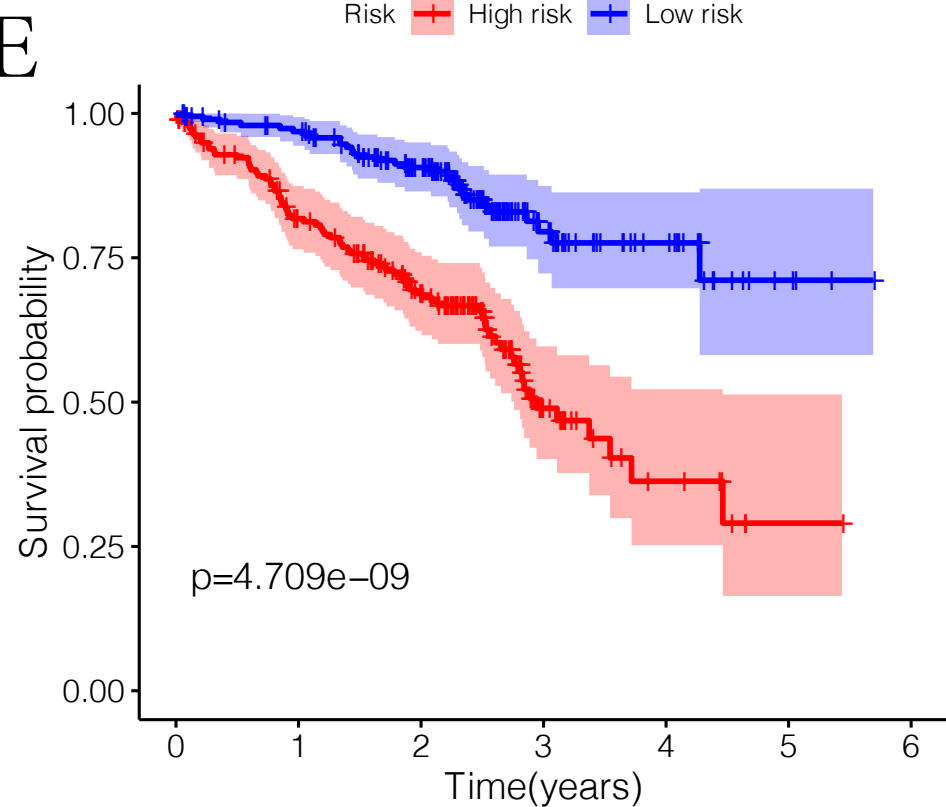

Supplement: Supplementary 2 — Figure S2: survival analysis. (A) TCGA-LUAD. (B) GSE30219. (C) GSE41271. (D) GSE50081. (E) GSE72094. [file 6567916.f2.pdf]

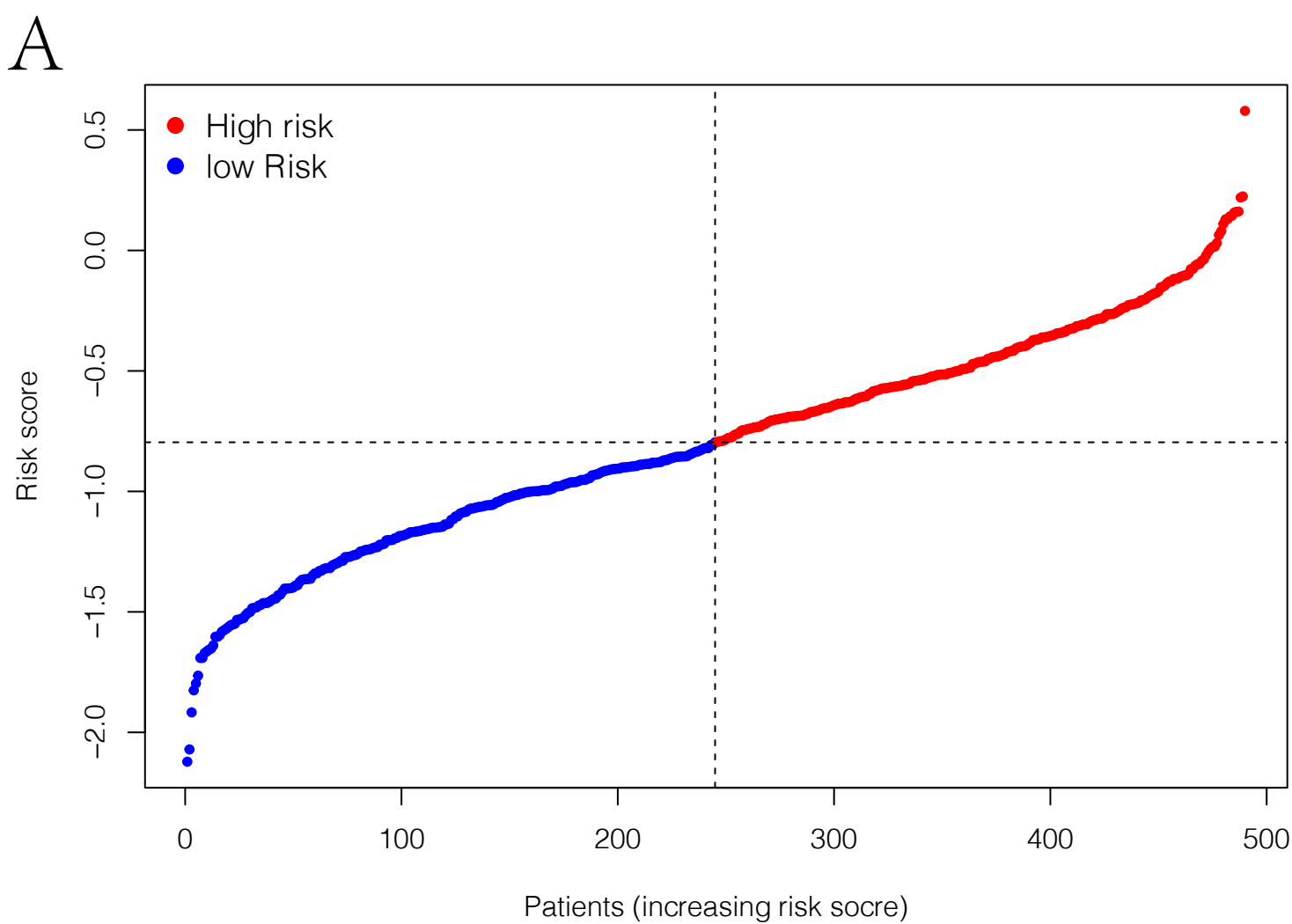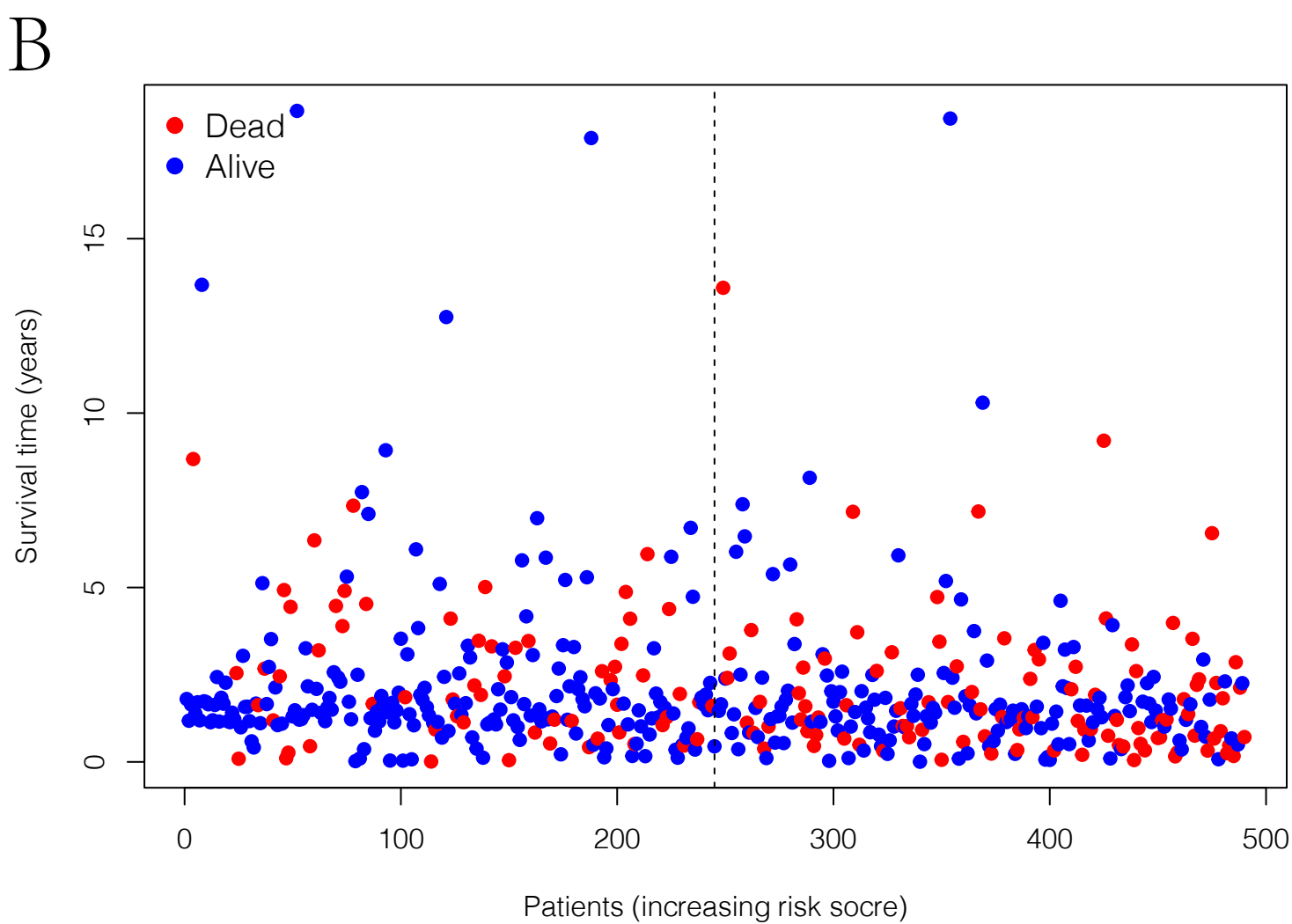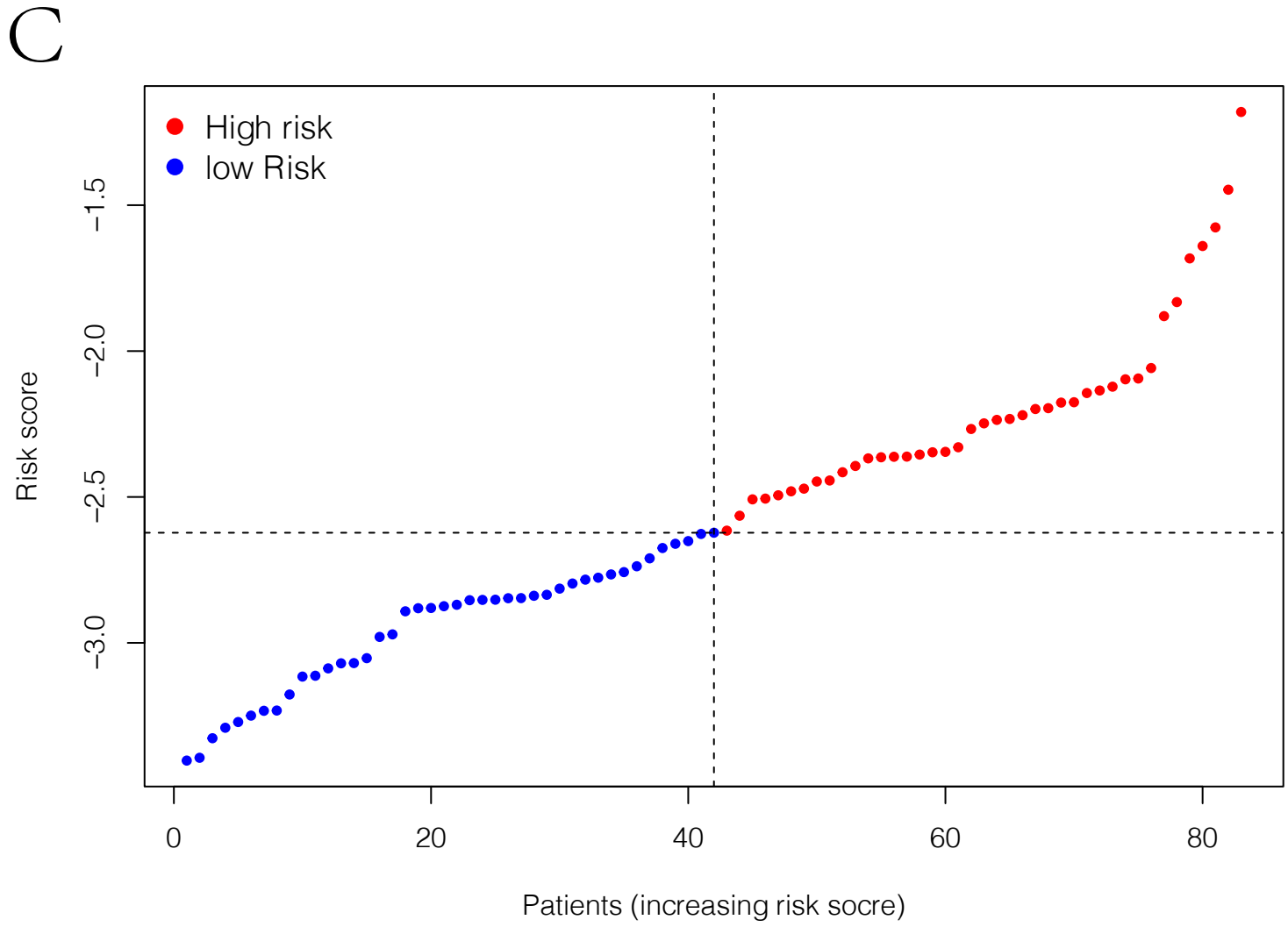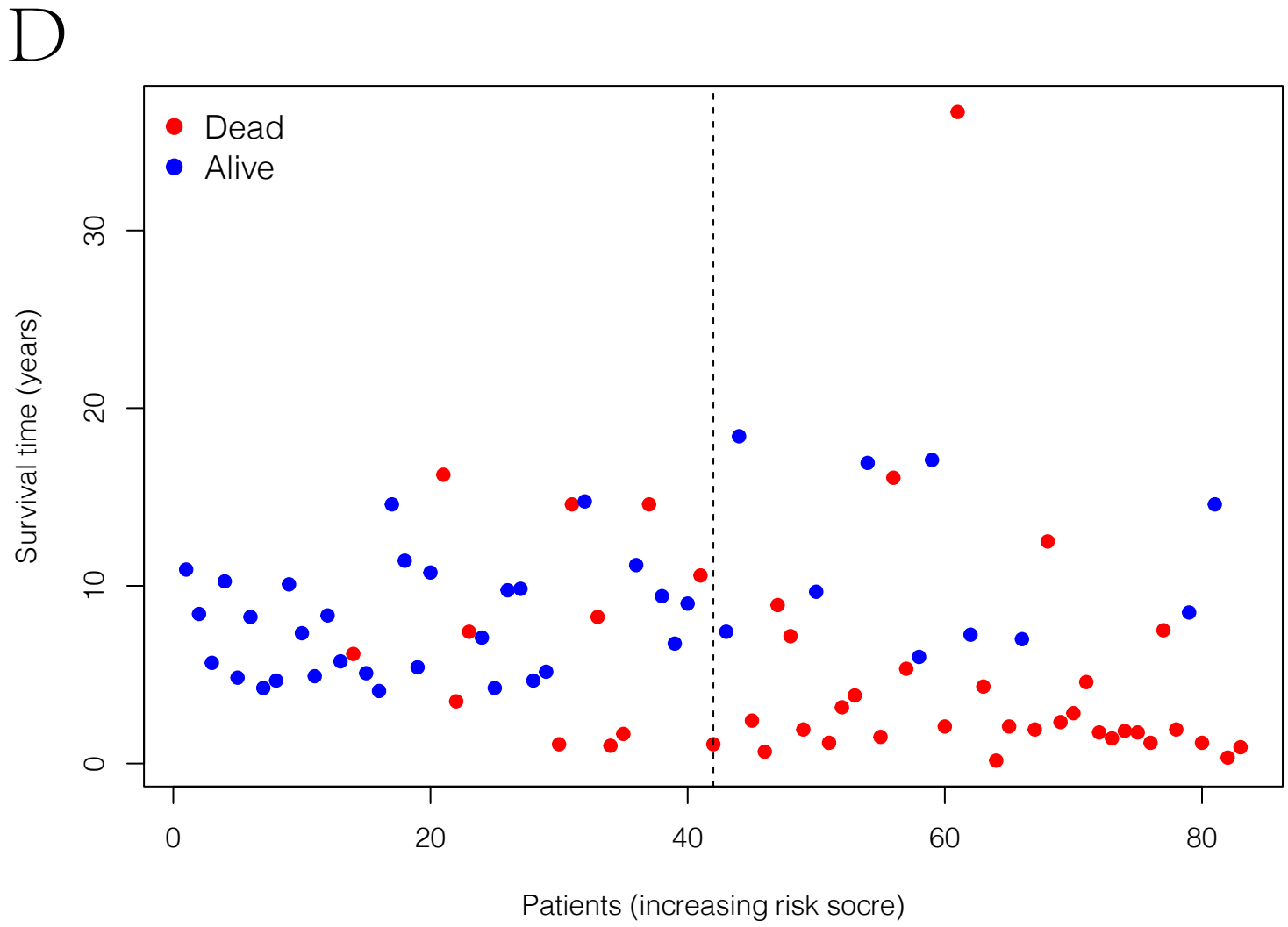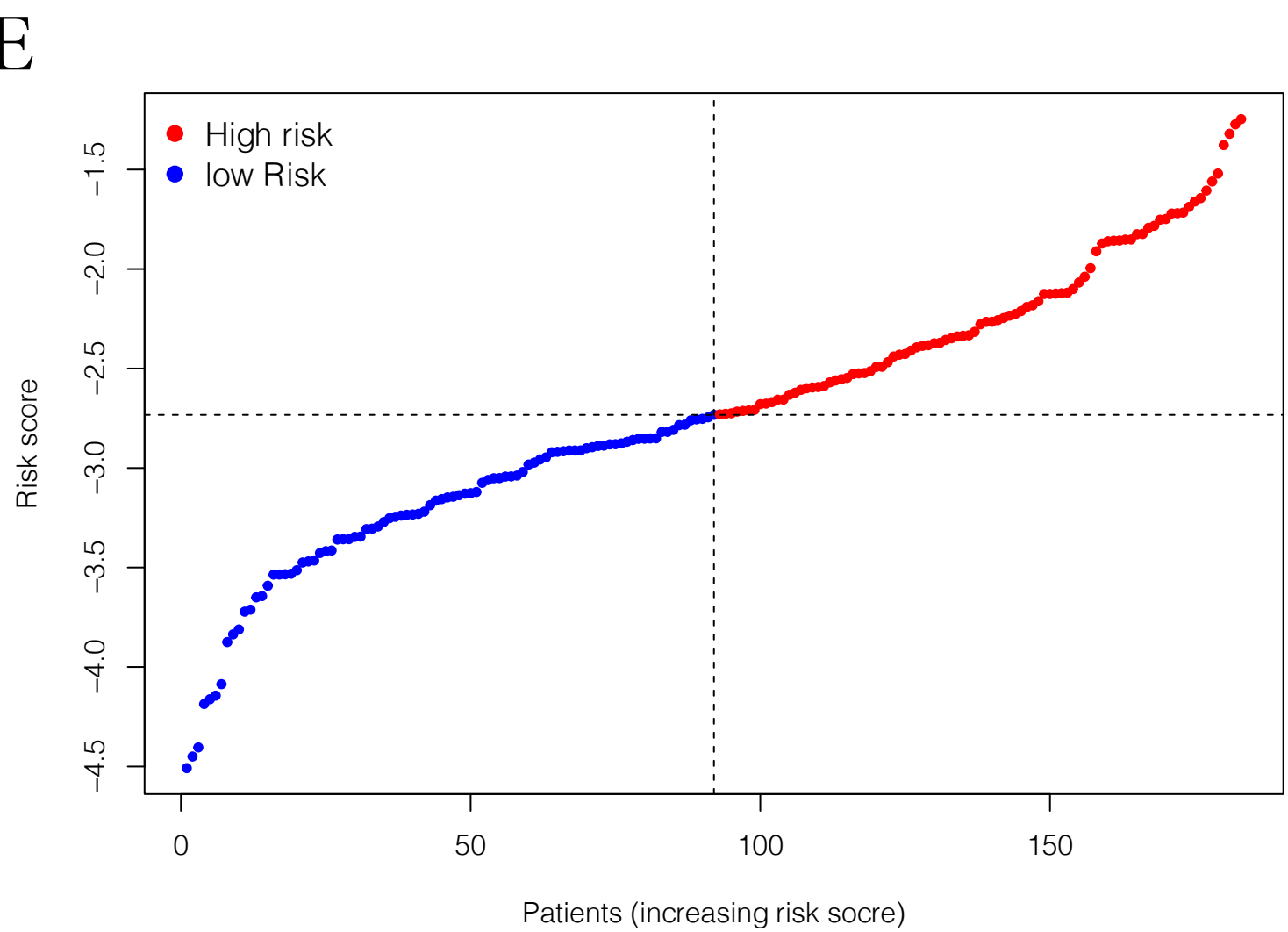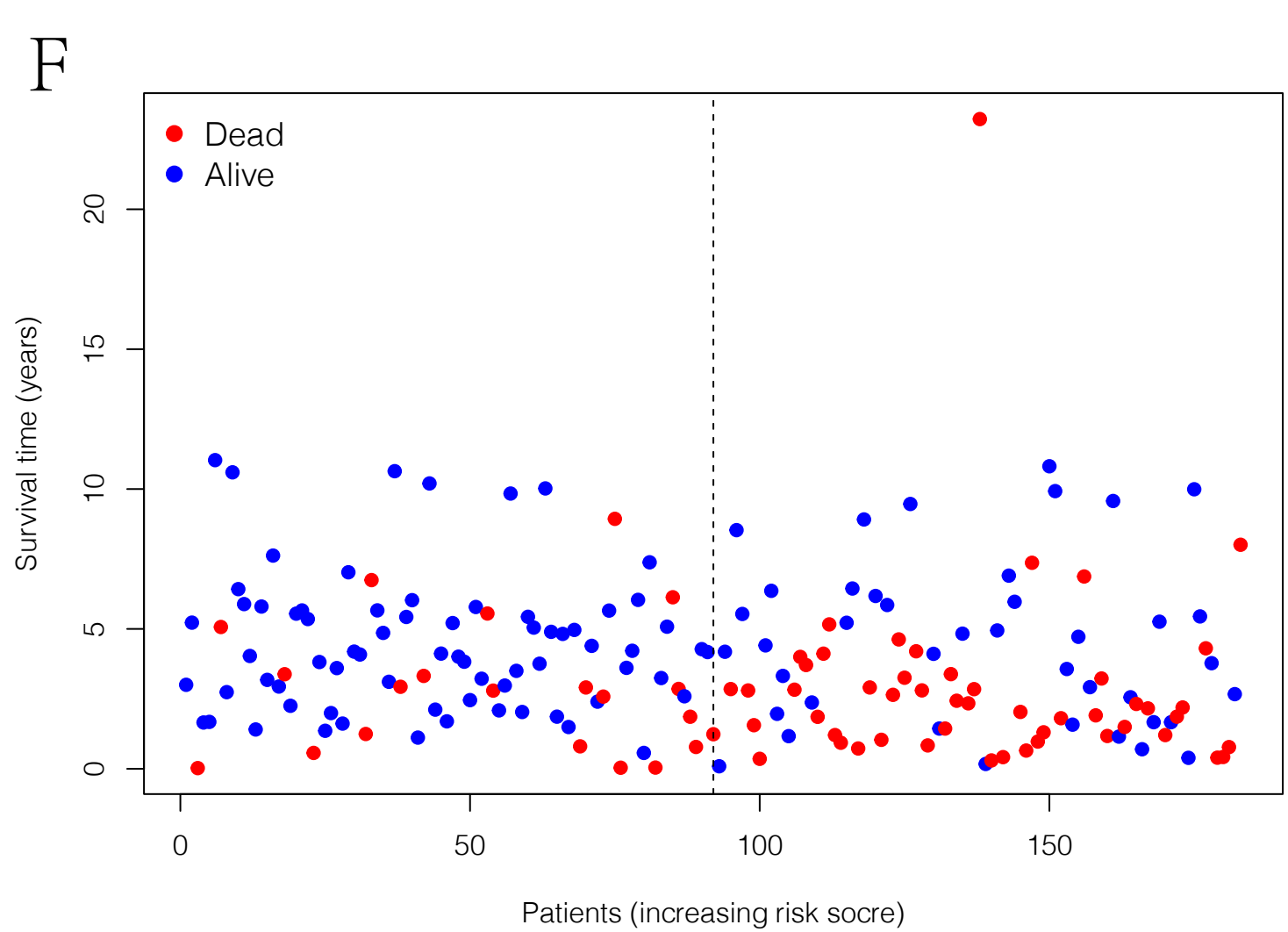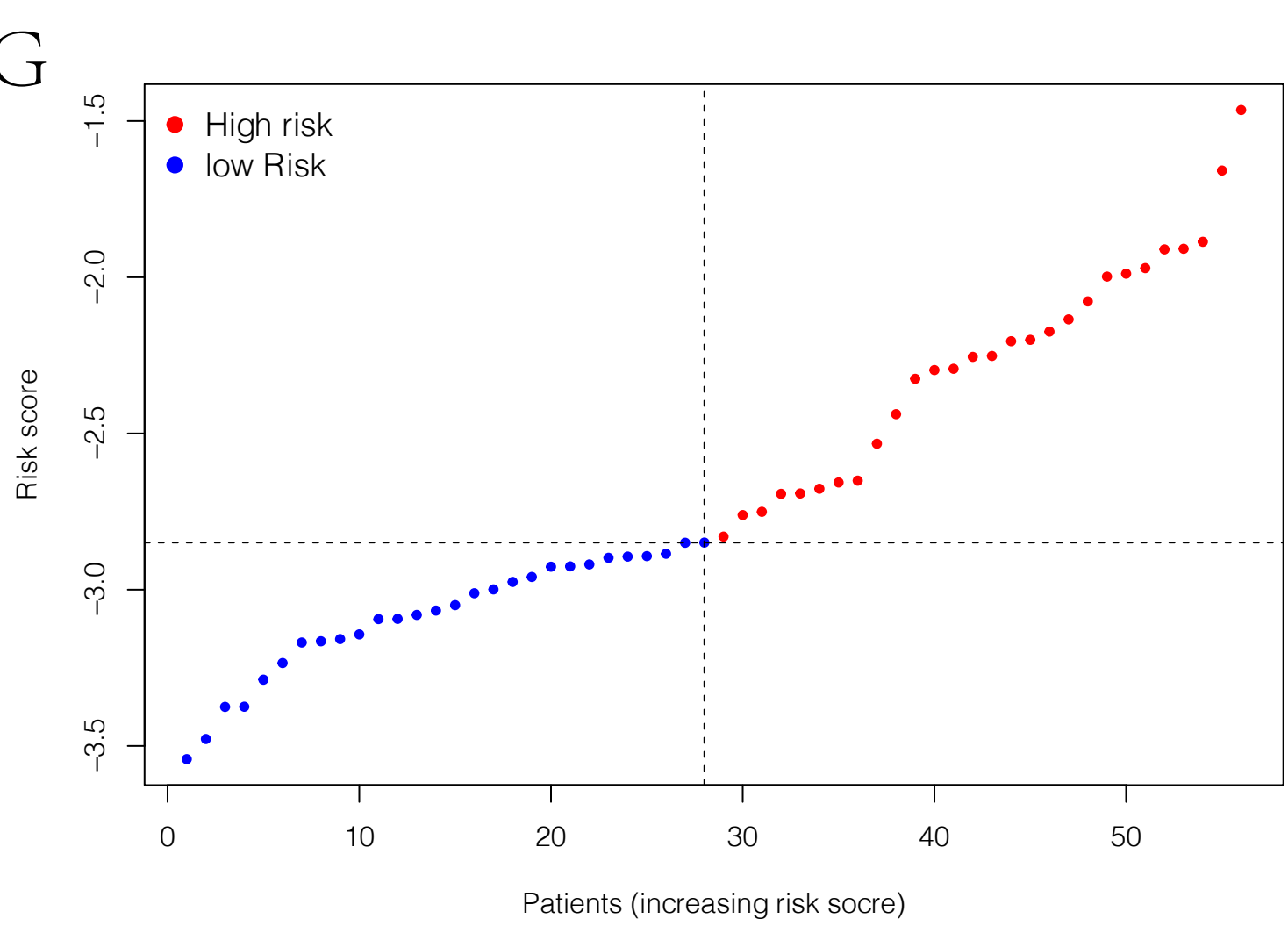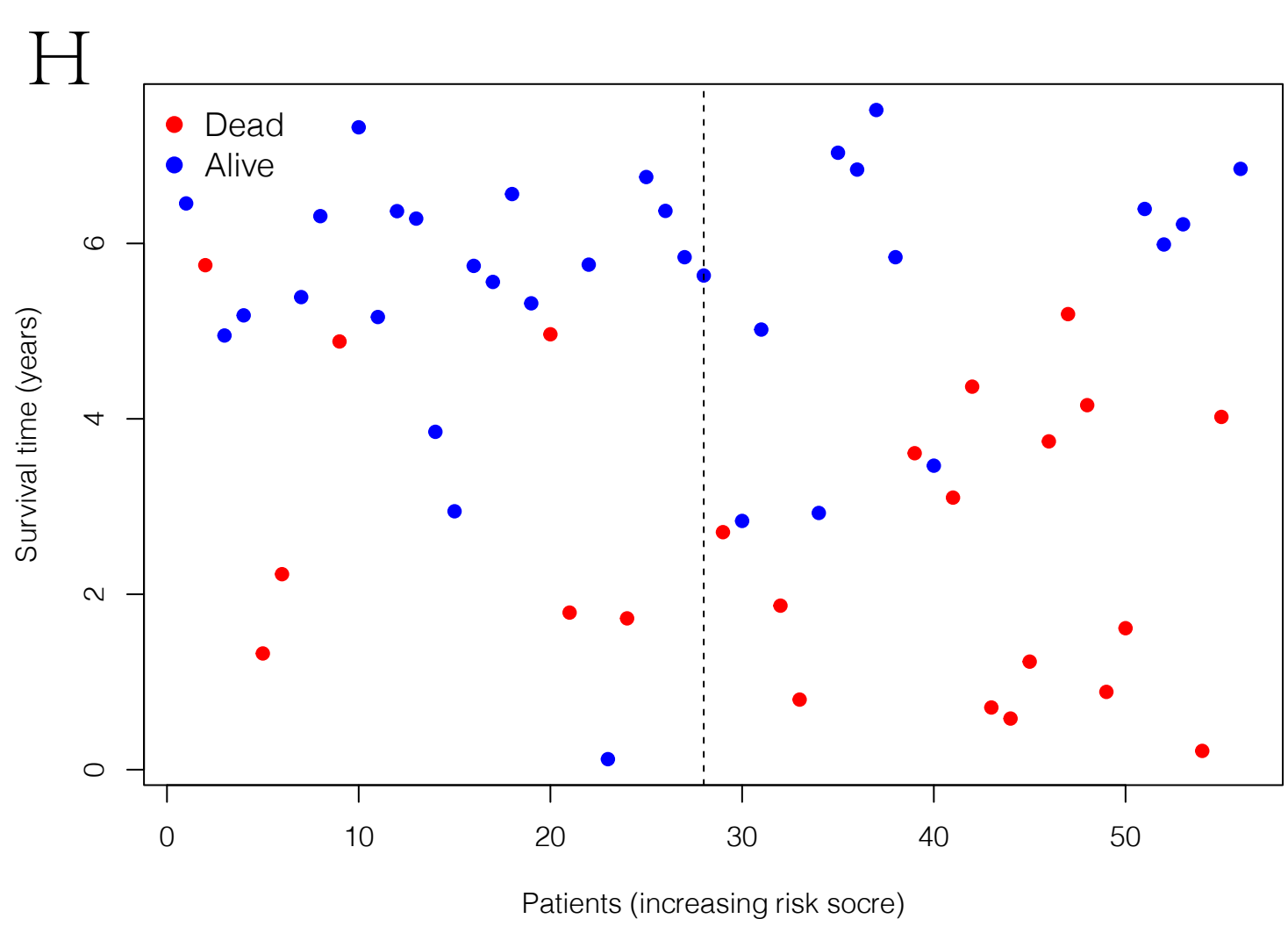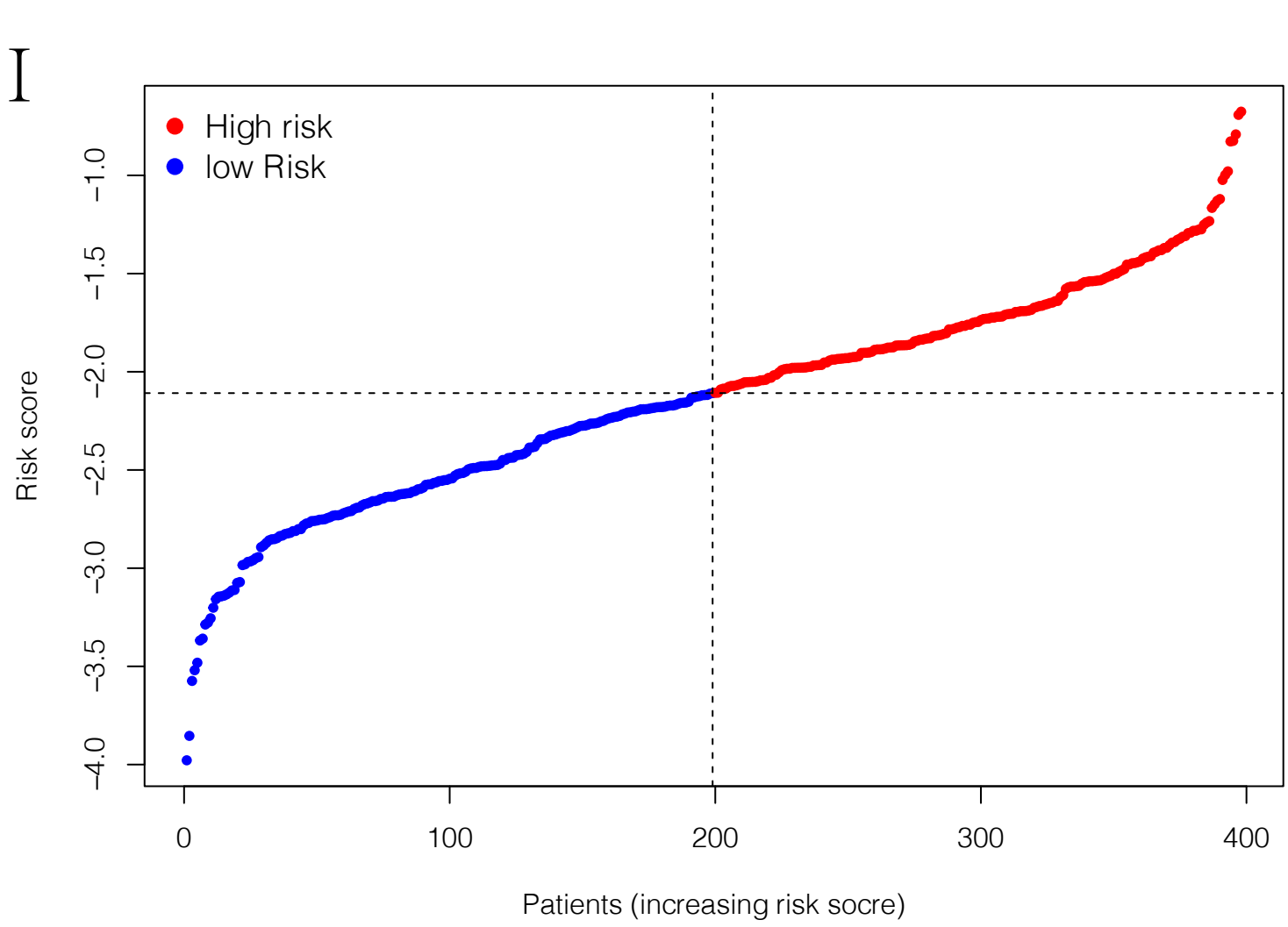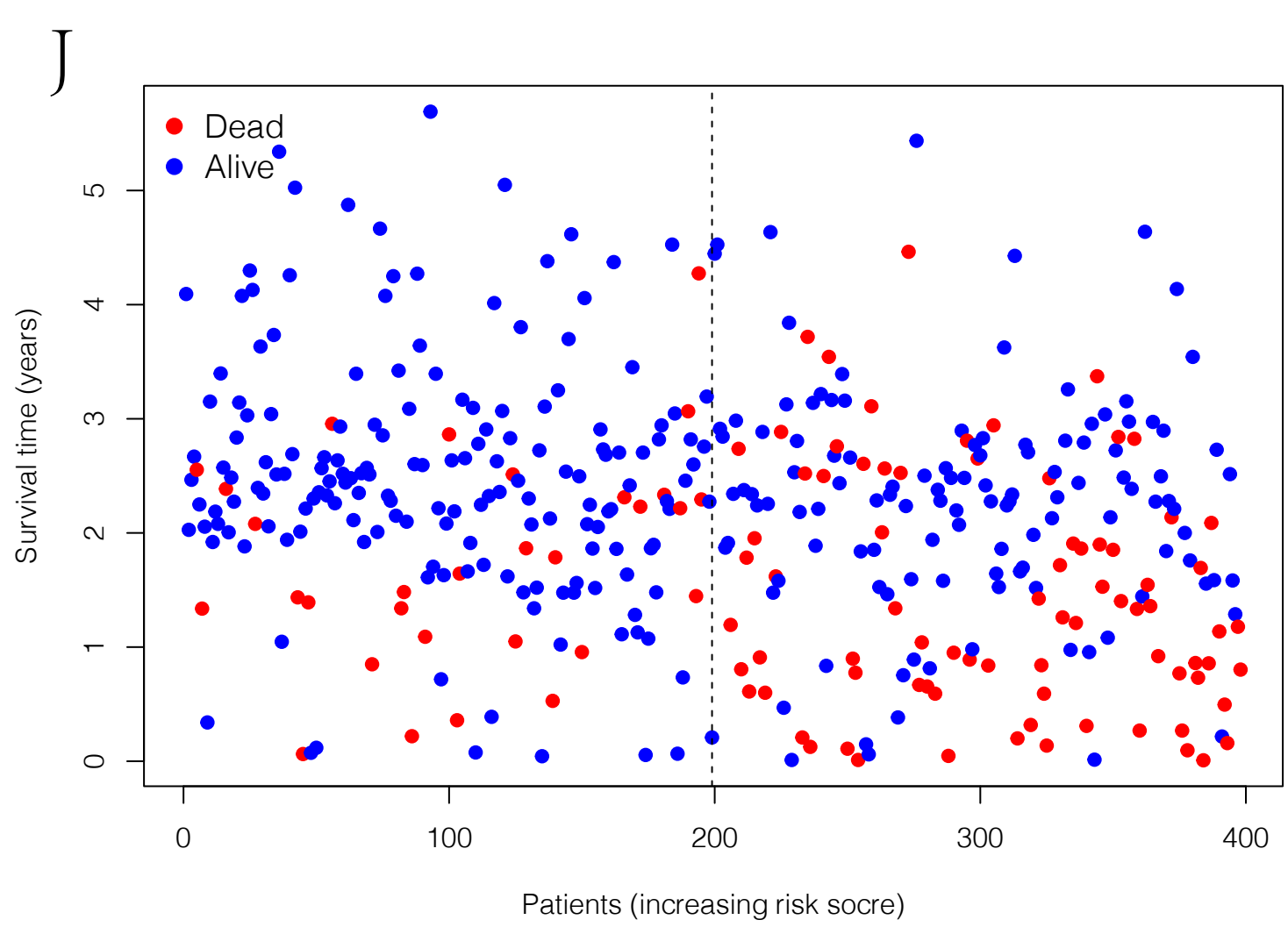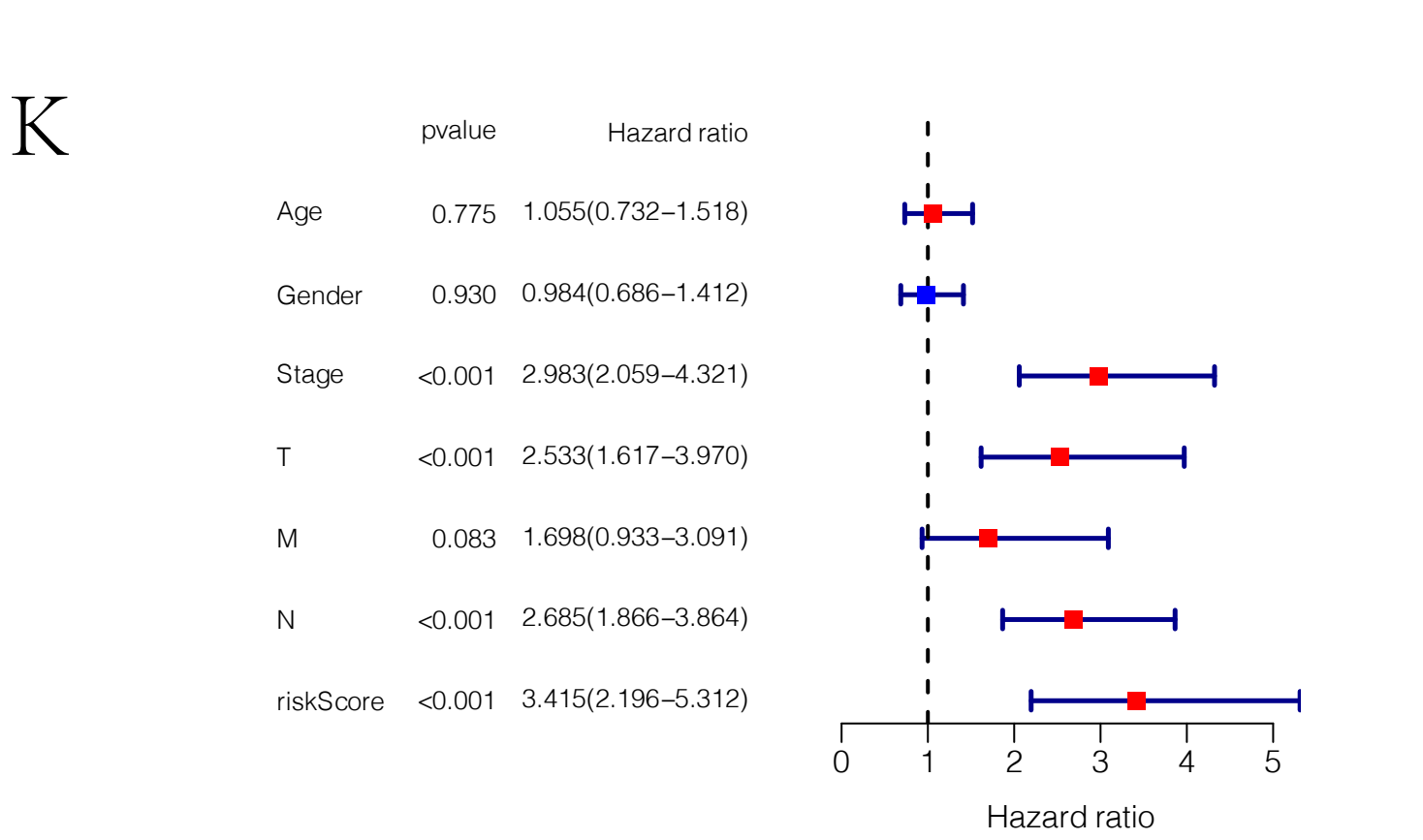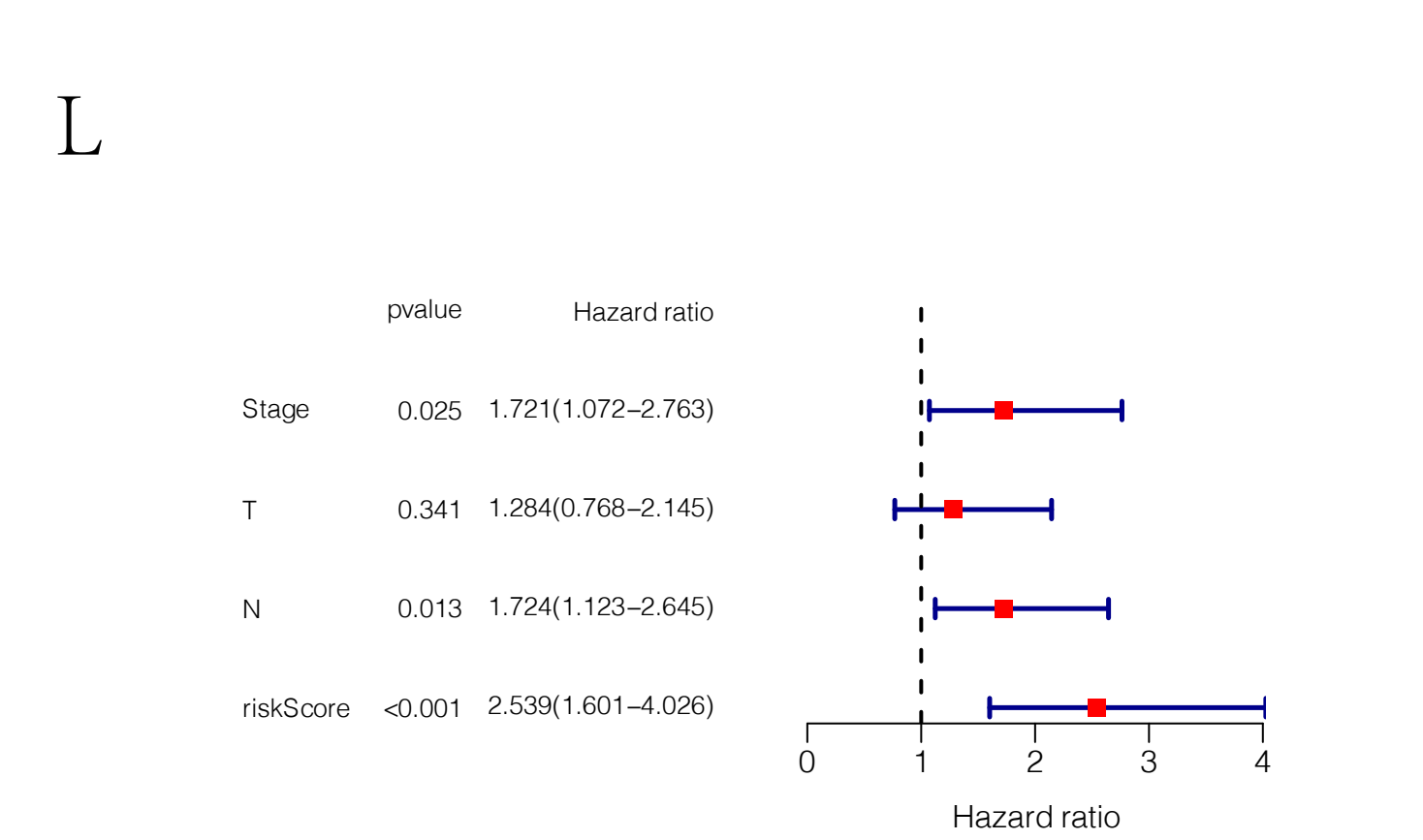

Supplement: Supplementary 3 — Figure S3: independence analysis, distribution of risk score, and scatterplots of the correlation between risk scores and survival time. [file 6567916.f3.pdf]

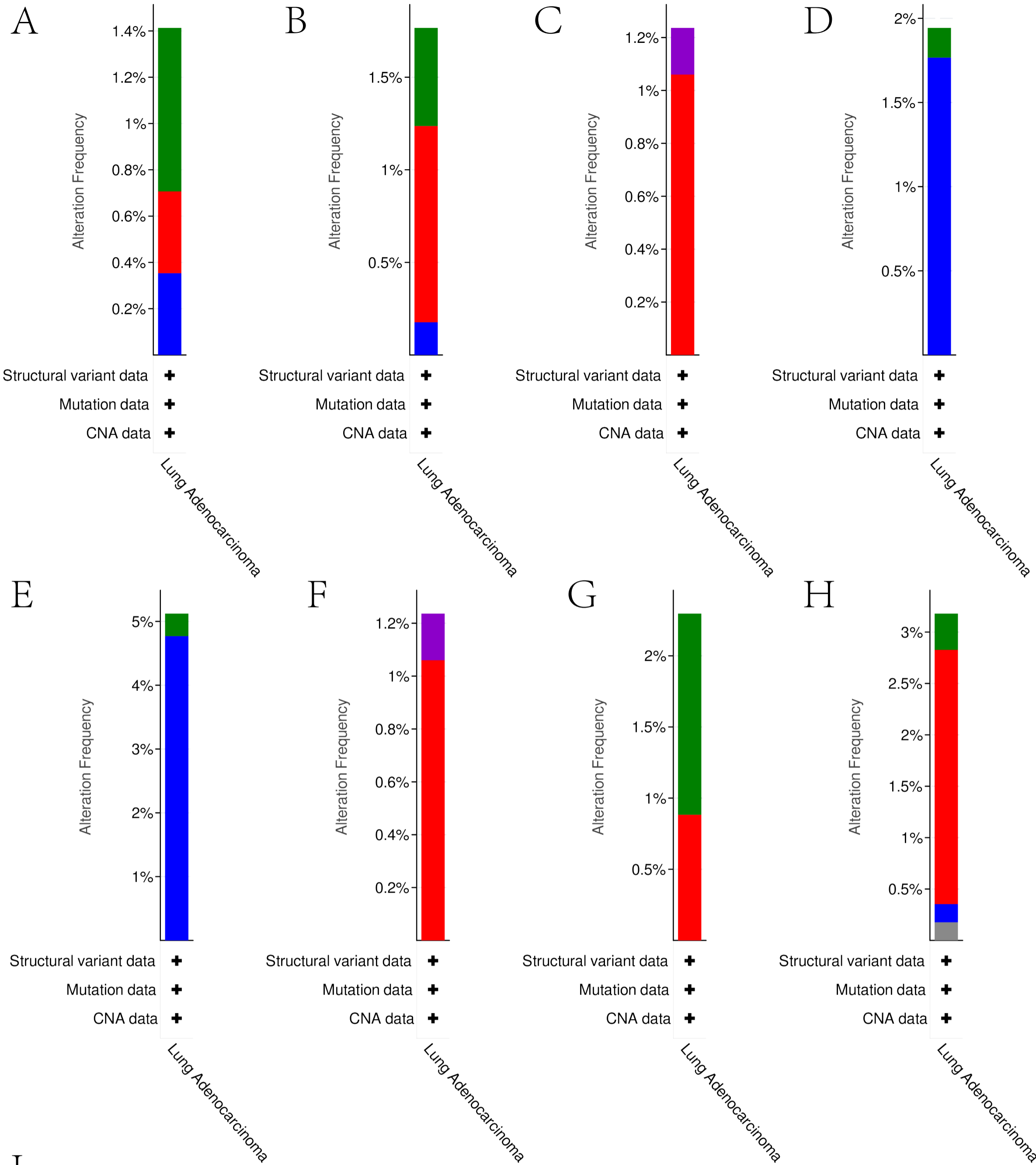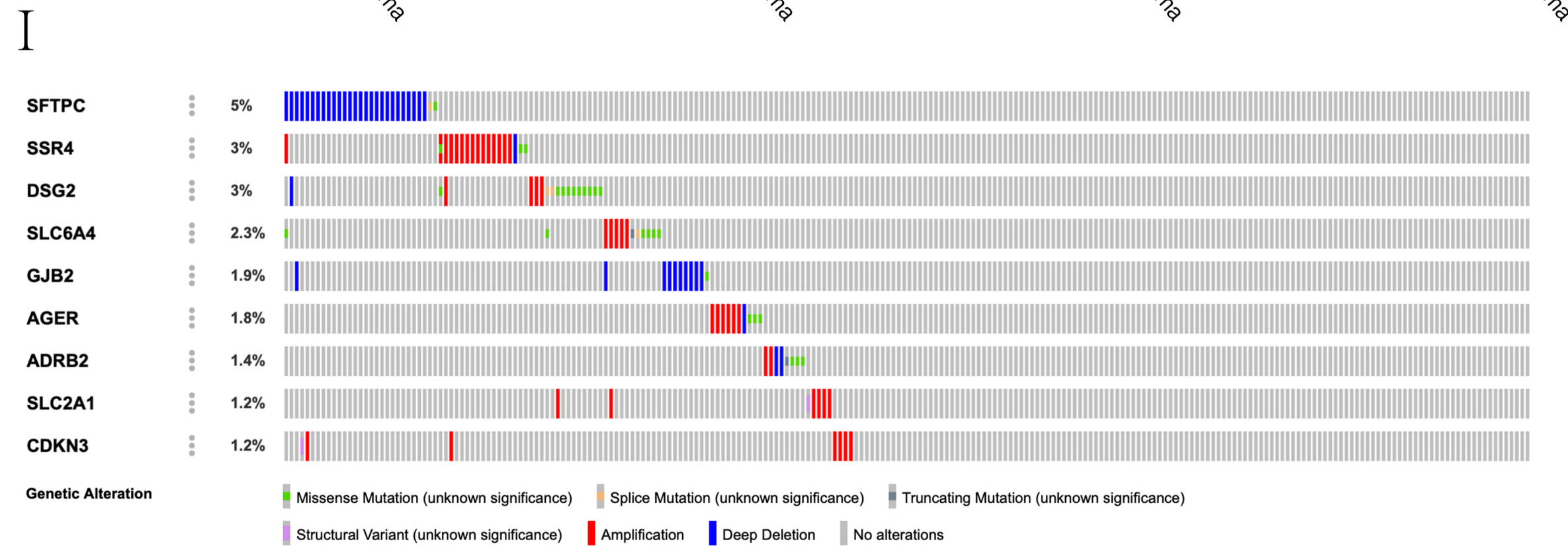

Supplement: Supplementary 4 — Figure S4: gene mutation. (A) ADRB2. (B) AGER. (C) CDKN3. (D) GJB2. (E) SFTPC. (F) SLC2A1. (G) SLC6A4. (H) SSR4. (I) Mutation summary of eight genes. [file 6567916.f4.pdf]
