# Supplementary material for: Prognostic Risk Signature and Comprehensive Analyses of Endoplasmic Reticulum Stress-Related Genes in Lung Adenocarcinoma
Source: J Immunol Res. 2022 May 4;2022:6567916. doi: 10.1155/2022/6567916 (PMC9096573; doi:10.1155/2022/6567916)
Supplement: Supplementary 5 — Table S1: ERS-related genes obtained from the previous literature (total 785). [file 6567916.f5.docx]

| Table S1 ERS-related genes obtained from the previous literature (total 785). | | |
| --- | --- | --- |
| Gene Symbol | Description | |
| HSPA5 | Heat Shock Protein Family A (Hsp70) Member 5 | |
| EIF2AK3 | Eukaryotic Translation Initiation Factor 2 Alpha Kinase 3 | |
| XBP1 | X-Box Binding Protein 1 | |
| ERN1 | Endoplasmic Reticulum To Nucleus Signaling 1 | |
| ATP2A2 | ATPase Sarcoplasmic/Endoplasmic Reticulum Ca2+ Transporting 2 | |
| ATF6 | Activating Transcription Factor 6 | |
| TP53 | Tumor Protein P53 | |
| VCP | Valosin Containing Protein | |
| HERPUD1 | Homocysteine Inducible ER Protein With Ubiquitin Like Domain 1 | |
| SOD1 | Superoxide Dismutase 1 | |
| RYR2 | Ryanodine Receptor 2 | |
| ATP2A1 | ATPase Sarcoplasmic/Endoplasmic Reticulum Ca2+ Transporting 1 | |
| HSP90B1 | Heat Shock Protein 90 Beta Family Member 1 | |
| TNF | Tumor Necrosis Factor | |
| EIF2S1 | Eukaryotic Translation Initiation Factor 2 Subunit Alpha | |
| PRKN | Parkin RBR E3 Ubiquitin Protein Ligase | |
| ATF4 | Activating Transcription Factor 4 | |
| DDIT3 | DNA Damage Inducible Transcript 3 | |
| APP | Amyloid Beta Precursor Protein | |
| PSEN1 | Presenilin 1 | |
| CANX | Calnexin |  |
| SYVN1 | Synoviolin 1 | |
| CALR | Calreticulin | |
| DNAJC3 | DnaJ Heat Shock Protein Family (Hsp40) Member C3 | |
| HYOU1 | Hypoxia Up-Regulated 1 | |
| ATP2A3 | ATPase Sarcoplasmic/Endoplasmic Reticulum Ca2+ Transporting 3 | |
| CAT | Catalase |  |
| INS | Insulin |  |
| SAR1B | Secretion Associated Ras Related GTPase 1B | |
| NFE2L2 | Nuclear Factor, Erythroid 2 Like 2 | |
| NFE2L1 | Nuclear Factor, Erythroid 2 Like 1 | |
| RYR1 | Ryanodine Receptor 1 | |
| DNAJC10 | DnaJ Heat Shock Protein Family (Hsp40) Member C10 | |
| ITPR1 | Inositol 1,4,5-Trisphosphate Receptor Type 1 | |
| CACNA1C | Calcium Voltage-Gated Channel Subunit Alpha1 C | |
| WFS1 | Wolframin ER Transmembrane Glycoprotein | |
| PPP1R15A | Protein Phosphatase 1 Regulatory Subunit 15A | |
| P4HB | Prolyl 4-Hydroxylase Subunit Beta | |
| CFTR | CF Transmembrane Conductance Regulator | |
| PRNP | Prion Protein | |
| MAPK8 | Mitogen-Activated Protein Kinase 8 | |
| ERO1A | Endoplasmic Reticulum Oxidoreductase 1 Alpha | |
| OS9 | OS9 Endoplasmic Reticulum Lectin | |
| DERL2 | Derlin 2 |  |
| BCL2 | BCL2 Apoptosis Regulator | |
| ERP29 | Endoplasmic Reticulum Protein 29 | |
| SIL1 | SIL1 Nucleotide Exchange Factor | |
| CLU | Clusterin |  |
| SERP1 | Stress Associated Endoplasmic Reticulum Protein 1 | |
| MAPT | Microtubule Associated Protein Tau | |
| PDIA3 | Protein Disulfide Isomerase Family A Member 3 | |
| TARDBP | TAR DNA Binding Protein | |
| MPO | Myeloperoxidase | |
| MAP3K5 | Mitogen-Activated Protein Kinase Kinase Kinase 5 | |
| H6PD | Hexose-6-Phosphate Dehydrogenase/Glucose 1-Dehydrogenase | |
| SERPINA1 | Serpin Family A Member 1 | |
| HSPA4 | Heat Shock Protein Family A (Hsp70) Member 4 | |
| CASQ1 | Calsequestrin 1 | |
| ERP44 | Endoplasmic Reticulum Protein 44 | |
| DERL1 | Derlin 1 |  |
| CASP4 | Caspase 4 |  |
| EPM2A | EPM2A Glucan Phosphatase, Laforin | |
| CXCL8 | C-X-C Motif Chemokine Ligand 8 | |
| CASP3 | Caspase 3 |  |
| NHLRC1 | NHL Repeat Containing E3 Ubiquitin Protein Ligase 1 | |
| BAX | BCL2 Associated X, Apoptosis Regulator | |
| ERAP1 | Endoplasmic Reticulum Aminopeptidase 1 | |
| STIM1 | Stromal Interaction Molecule 1 | |
| DSP | Desmoplakin | |
| HLA-DRB1 | Major Histocompatibility Complex, Class II, DR Beta 1 | |
| F2 | Coagulation Factor II, Thrombin | |
| HMOX1 | Heme Oxygenase 1 | |
| KDELR1 | KDEL Endoplasmic Reticulum Protein Retention Receptor 1 | |
| MIA2 | MIA SH3 Domain ER Export Factor 2 | |
| SNCA | Synuclein Alpha | |
| IL1B | Interleukin 1 Beta | |
| DERL3 | Derlin 3 |  |
| SEC16A | SEC16 Homolog A, Endoplasmic Reticulum Export Factor | |
| MBTPS2 | Membrane Bound Transcription Factor Peptidase, Site 2 | |
| ERLEC1 | Endoplasmic Reticulum Lectin 1 | |
| SEC23A | Sec23 Homolog A, COPII Coat Complex Component | |
| AMFR | Autocrine Motility Factor Receptor | |
| MANF | Mesencephalic Astrocyte Derived Neurotrophic Factor | |
| SERP2 | Stress Associated Endoplasmic Reticulum Protein Family Member 2 | |
| SEL1L | SEL1L Adaptor Subunit Of ERAD E3 Ubiquitin Ligase | |
| APOB | Apolipoprotein B | |
| ALB | Albumin |  |
| TRDN | Triadin |  |
| HSP90AA1 | Heat Shock Protein 90 Alpha Family Class A Member 1 | |
| LMAN1 | Lectin, Mannose Binding 1 | |
| HSPA1A | Heat Shock Protein Family A (Hsp70) Member 1A | |
| CDKN3 | Cyclin Dependent Kinase Inhibitor 3 | |
| ERN2 | Endoplasmic Reticulum To Nucleus Signaling 2 | |
| SOD2 | Superoxide Dismutase 2 | |
| PDIA4 | Protein Disulfide Isomerase Family A Member 4 | |
| POMC | Proopiomelanocortin | |
| ALG2 | ALG2 Alpha-1,3/1,6-Mannosyltransferase | |
| SELENOS | Selenoprotein S | |
| CREB3L1 | CAMP Responsive Element Binding Protein 3 Like 1 | |
| APOE | Apolipoprotein E | |
| HSPA8 | Heat Shock Protein Family A (Hsp70) Member 8 | |
| HSF1 | Heat Shock Transcription Factor 1 | |
| KCNH2 | Potassium Voltage-Gated Channel Subfamily H Member 2 | |
| HFE | Homeostatic Iron Regulator | |
| EIF2AK4 | Eukaryotic Translation Initiation Factor 2 Alpha Kinase 4 | |
| MAPK14 | Mitogen-Activated Protein Kinase 14 | |
| COMT | Catechol-O-Methyltransferase | |
| GFAP | Glial Fibrillary Acidic Protein | |
| ELN | Elastin |  |
| CYP21A2 | Cytochrome P450 Family 21 Subfamily A Member 2 | |
| KDELR2 | KDEL Endoplasmic Reticulum Protein Retention Receptor 2 | |
| SEC23B | SEC23 Homolog B, COPII Coat Complex Component | |
| BCAP31 | B Cell Receptor Associated Protein 31 | |
| CD4 | CD4 Molecule | |
| PSEN2 | Presenilin 2 | |
| MAN1B1 | Mannosidase Alpha Class 1B Member 1 | |
| EDN1 | Endothelin 1 | |
| DMD | Dystrophin | |
| DRD5 | Dopamine Receptor D5 | |
| SEC13 | SEC13 Homolog, Nuclear Pore And COPII Coat Complex Component | |
| ERAP2 | Endoplasmic Reticulum Aminopeptidase 2 | |
| ATF6B | Activating Transcription Factor 6 Beta | |
| RPS27A | Ribosomal Protein S27a | |
| BAG6 | BAG Cochaperone 6 | |
| SPP1 | Secreted Phosphoprotein 1 | |
| DNAJB9 | DnaJ Heat Shock Protein Family (Hsp40) Member B9 | |
| COMP | Cartilage Oligomeric Matrix Protein | |
| JUP | Junction Plakoglobin | |
| ANK2 | Ankyrin 2 |  |
| CREB3 | CAMP Responsive Element Binding Protein 3 | |
| MOGS | Mannosyl-Oligosaccharide Glucosidase | |
| SLC26A2 | Solute Carrier Family 26 Member 2 | |
| SEC61A1 | SEC61 Translocon Subunit Alpha 1 | |
| CR2 | Complement C3d Receptor 2 | |
| CYCS | Cytochrome C, Somatic | |
| SNAP25 | Synaptosome Associated Protein 25 | |
| MAPK1 | Mitogen-Activated Protein Kinase 1 | |
| SERPINH1 | Serpin Family H Member 1 | |
| SCN5A | Sodium Voltage-Gated Channel Alpha Subunit 5 | |
| JUN | Jun Proto-Oncogene, AP-1 Transcription Factor Subunit | |
| EDNRA | Endothelin Receptor Type A | |
| PDIA2 | Protein Disulfide Isomerase Family A Member 2 | |
| SAR1A | Secretion Associated Ras Related GTPase 1A | |
| DNAJB11 | DnaJ Heat Shock Protein Family (Hsp40) Member B11 | |
| F9 | Coagulation Factor IX | |
| DNAJB1 | DnaJ Heat Shock Protein Family (Hsp40) Member B1 | |
| TXNDC12 | Thioredoxin Domain Containing 12 | |
| SCN2A | Sodium Voltage-Gated Channel Alpha Subunit 2 | |
| NOS3 | Nitric Oxide Synthase 3 | |
| CISD2 | CDGSH Iron Sulfur Domain 2 | |
| EDEM1 | ER Degradation Enhancing Alpha-Mannosidase Like Protein 1 | |
| GLA | Galactosidase Alpha | |
| SLC6A4 | Solute Carrier Family 6 Member 4 | |
| SEC24C | SEC24 Homolog C, COPII Coat Complex Component | |
| ERO1B | Endoplasmic Reticulum Oxidoreductase 1 Beta | |
| SELENOK | Selenoprotein K | |
| PTPN1 | Protein Tyrosine Phosphatase Non-Receptor Type 1 | |
| SEC24B | SEC24 Homolog B, COPII Coat Complex Component | |
| PLN | Phospholamban | |
| HTT | Huntingtin |  |
| SP1 | Sp1 Transcription Factor | |
| EIF2AK2 | Eukaryotic Translation Initiation Factor 2 Alpha Kinase 2 | |
| GJB2 | Gap Junction Protein Beta 2 | |
| MTTP | Microsomal Triglyceride Transfer Protein | |
| UBC | Ubiquitin C | |
| FOS | Fos Proto-Oncogene, AP-1 Transcription Factor Subunit | |
| CASP8 | Caspase 8 |  |
| RTN4 | Reticulon 4 | |
| CREB3L2 | CAMP Responsive Element Binding Protein 3 Like 2 | |
| SIRT1 | Sirtuin 1 |  |
| CRH | Corticotropin Releasing Hormone | |
| KCNJ11 | Potassium Inwardly Rectifying Channel Subfamily J Member 11 | |
| CASP9 | Caspase 9 |  |
| CREB3L3 | CAMP Responsive Element Binding Protein 3 Like 3 | |
| TXNDC5 | Thioredoxin Domain Containing 5 | |
| VAPB | VAMP Associated Protein B And C | |
| PARK7 | Parkinsonism Associated Deglycase | |
| ERP27 | Endoplasmic Reticulum Protein 27 | |
| TOR1A | Torsin Family 1 Member A | |
| RTN3 | Reticulon 3 | |
| SIGMAR1 | Sigma Non-Opioid Intracellular Receptor 1 | |
| PARP1 | Poly(ADP-Ribose) Polymerase 1 | |
| IL6 | Interleukin 6 | |
| UBQLN1 | Ubiquilin 1 | |
| DSC2 | Desmocollin 2 | |
| LMNA | Lamin A/C | |
| TH | Tyrosine Hydroxylase | |
| SERPINA3 | Serpin Family A Member 3 | |
| RPN1 | Ribophorin I | |
| LRRK2 | Leucine Rich Repeat Kinase 2 | |
| TF | Transferrin | |
| INPP5K | Inositol Polyphosphate-5-Phosphatase K | |
| APOA1 | Apolipoprotein A1 | |
| RER1 | Retention In Endoplasmic Reticulum Sorting Receptor 1 | |
| UGGT1 | UDP-Glucose Glycoprotein Glucosyltransferase 1 | |
| TXN | Thioredoxin | |
| ORAI1 | ORAI Calcium Release-Activated Calcium Modulator 1 | |
| CSTB | Cystatin B |  |
| CHERP | Calcium Homeostasis Endoplasmic Reticulum Protein | |
| MAPK10 | Mitogen-Activated Protein Kinase 10 | |
| BAK1 | BCL2 Antagonist/Killer 1 | |
| GJA1 | Gap Junction Protein Alpha 1 | |
| PPP1R15B | Protein Phosphatase 1 Regulatory Subunit 15B | |
| AKT1 | AKT Serine/Threonine Kinase 1 | |
| C1S | Complement C1s | |
| SEC31A | SEC31 Homolog A, COPII Coat Complex Component | |
| PTGS2 | Prostaglandin-Endoperoxide Synthase 2 | |
| PPIB | Peptidylprolyl Isomerase B | |
| MTOR | Mechanistic Target Of Rapamycin Kinase | |
| LGI4 | Leucine Rich Repeat LGI Family Member 4 | |
| GSR | Glutathione-Disulfide Reductase | |
| ALG1 | ALG1 Chitobiosyldiphosphodolichol Beta-Mannosyltransferase | |
| DPM1 | Dolichyl-Phosphate Mannosyltransferase Subunit 1, Catalytic | |
| KCNE1 | Potassium Voltage-Gated Channel Subfamily E Regulatory Subunit 1 | |
| DNAH8 | Dynein Axonemal Heavy Chain 8 | |
| TRAF2 | TNF Receptor Associated Factor 2 | |
| CIB1 | Calcium And Integrin Binding 1 | |
| AVP | Arginine Vasopressin | |
| VEGFA | Vascular Endothelial Growth Factor A | |
| DDOST | Dolichyl-Diphosphooligosaccharide--Protein Glycosyltransferase Non-Catalytic Subunit | |
| SESN2 | Sestrin 2 |  |
| C1R | Complement C1r | |
| IL7 | Interleukin 7 | |
| LMAN2 | Lectin, Mannose Binding 2 | |
| ATF3 | Activating Transcription Factor 3 | |
| SSR4 | Signal Sequence Receptor Subunit 4 | |
| HMGCR | 3-Hydroxy-3-Methylglutaryl-CoA Reductase | |
| NOS2 | Nitric Oxide Synthase 2 | |
| IGF2R | Insulin Like Growth Factor 2 Receptor | |
| ESYT1 | Extended Synaptotagmin 1 | |
| UBE2J1 | Ubiquitin Conjugating Enzyme E2 J1 | |
| TFRC | Transferrin Receptor | |
| LNPK | Lunapark, ER Junction Formation Factor | |
| HSPB1 | Heat Shock Protein Family B (Small) Member 1 | |
| TRPV4 | Transient Receptor Potential Cation Channel Subfamily V Member 4 | |
| COL2A1 | Collagen Type II Alpha 1 Chain | |
| VWF | Von Willebrand Factor | |
| RNF185 | Ring Finger Protein 185 | |
| HP | Haptoglobin | |
| IAPP | Islet Amyloid Polypeptide | |
| SERPINC1 | Serpin Family C Member 1 | |
| BDNF | Brain Derived Neurotrophic Factor | |
| NOX4 | NADPH Oxidase 4 | |
| HSD11B1 | Hydroxysteroid 11-Beta Dehydrogenase 1 | |
| ITPR3 | Inositol 1,4,5-Trisphosphate Receptor Type 3 | |
| TMBIM6 | Transmembrane BAX Inhibitor Motif Containing 6 | |
| SEC62 | SEC62 Homolog, Preprotein Translocation Factor | |
| EBP | EBP Cholestenol Delta-Isomerase | |
| TAP1 | Transporter 1, ATP Binding Cassette Subfamily B Member | |
| GATA1 | GATA Binding Protein 1 | |
| XDH | Xanthine Dehydrogenase | |
| CTNNB1 | Catenin Beta 1 | |
| SEC61B | SEC61 Translocon Subunit Beta | |
| SELENON | Selenoprotein N | |
| ITPR2 | Inositol 1,4,5-Trisphosphate Receptor Type 2 | |
| CASP12 | Caspase 12 (Gene/Pseudogene) | |
| TAPBP | TAP Binding Protein | |
| CKAP4 | Cytoskeleton Associated Protein 4 | |
| CLN3 | CLN3 Lysosomal/Endosomal Transmembrane Protein, Battenin | |
| CNGA3 | Cyclic Nucleotide Gated Channel Subunit Alpha 3 | |
| SLC8A1 | Solute Carrier Family 8 Member A1 | |
| THBS1 | Thrombospondin 1 | |
| BNIP1 | BCL2 Interacting Protein 1 | |
| EGFR | Epidermal Growth Factor Receptor | |
| SQSTM1 | Sequestosome 1 | |
| LPL | Lipoprotein Lipase | |
| STUB1 | STIP1 Homology And U-Box Containing Protein 1 | |
| HSPA1B | Heat Shock Protein Family A (Hsp70) Member 1B | |
| NR3C1 | Nuclear Receptor Subfamily 3 Group C Member 1 | |
| RPN2 | Ribophorin II | |
| AKAP9 | A-Kinase Anchoring Protein 9 | |
| CACNA1S | Calcium Voltage-Gated Channel Subunit Alpha1 S | |
| MYH7 | Myosin Heavy Chain 7 | |
| TGFB1 | Transforming Growth Factor Beta 1 | |
| NNT | Nicotinamide Nucleotide Transhydrogenase | |
| ADIPOQ | Adiponectin, C1Q And Collagen Domain Containing | |
| BCL2L1 | BCL2 Like 1 | |
| ACP1 | Acid Phosphatase 1 | |
| CST3 | Cystatin C |  |
| SURF4 | Surfeit 4 |  |
| PON1 | Paraoxonase 1 | |
| MBTPS1 | Membrane Bound Transcription Factor Peptidase, Site 1 | |
| NFKB1 | Nuclear Factor Kappa B Subunit 1 | |
| ABL1 | ABL Proto-Oncogene 1, Non-Receptor Tyrosine Kinase | |
| CCL2 | C-C Motif Chemokine Ligand 2 | |
| STING1 | Stimulator Of Interferon Response CGAMP Interactor 1 | |
| BBC3 | BCL2 Binding Component 3 | |
| CASQ2 | Calsequestrin 2 | |
| CRYAB | Crystallin Alpha B | |
| SEC63 | SEC63 Homolog, Protein Translocation Regulator | |
| ERLIN2 | ER Lipid Raft Associated 2 | |
| ELANE | Elastase, Neutrophil Expressed | |
| IGF2BP2 | Insulin Like Growth Factor 2 MRNA Binding Protein 2 | |
| MAPK9 | Mitogen-Activated Protein Kinase 9 | |
| VIM | Vimentin |  |
| TNFRSF10B | TNF Receptor Superfamily Member 10b | |
| HLA-B | Major Histocompatibility Complex, Class I, B | |
| INSIG1 | Insulin Induced Gene 1 | |
| BACE1 | Beta-Secretase 1 | |
| CEBPB | CCAAT Enhancer Binding Protein Beta | |
| PDCD6 | Programmed Cell Death 6 | |
| RRBP1 | Ribosome Binding Protein 1 | |
| CANT1 | Calcium Activated Nucleotidase 1 | |
| MAP2 | Microtubule Associated Protein 2 | |
| ERMP1 | Endoplasmic Reticulum Metallopeptidase 1 | |
| UGGT2 | UDP-Glucose Glycoprotein Glucosyltransferase 2 | |
| TRIM13 | Tripartite Motif Containing 13 | |
| VCL | Vinculin |  |
| BRSK2 | BR Serine/Threonine Kinase 2 | |
| CYBA | Cytochrome B-245 Alpha Chain | |
| CRYAA | Crystallin Alpha A | |
| GBF1 | Golgi Brefeldin A Resistant Guanine Nucleotide Exchange Factor 1 | |
| JPH2 | Junctophilin 2 | |
| TRIP11 | Thyroid Hormone Receptor Interactor 11 | |
| HSPA9 | Heat Shock Protein Family A (Hsp70) Member 9 | |
| PDIA6 | Protein Disulfide Isomerase Family A Member 6 | |
| ADAM10 | ADAM Metallopeptidase Domain 10 | |
| DYSF | Dysferlin |  |
| SREBF1 | Sterol Regulatory Element Binding Transcription Factor 1 | |
| TRPC1 | Transient Receptor Potential Cation Channel Subfamily C Member 1 | |
| KEAP1 | Kelch Like ECH Associated Protein 1 | |
| TLR4 | Toll Like Receptor 4 | |
| CYP2E1 | Cytochrome P450 Family 2 Subfamily E Member 1 | |
| SGK1 | Serum/Glucocorticoid Regulated Kinase 1 | |
| HSD11B2 | Hydroxysteroid 11-Beta Dehydrogenase 2 | |
| PPARG | Peroxisome Proliferator Activated Receptor Gamma | |
| TMEM33 | Transmembrane Protein 33 | |
| UBB | Ubiquitin B | |
| PMM2 | Phosphomannomutase 2 | |
| RTN1 | Reticulon 1 | |
| ESR1 | Estrogen Receptor 1 | |
| FAF2 | Fas Associated Factor Family Member 2 | |
| GET3 | Guided Entry Of Tail-Anchored Proteins Factor 3, ATPase | |
| HSP90AB1 | Heat Shock Protein 90 Alpha Family Class B Member 1 | |
| UBE2G2 | Ubiquitin Conjugating Enzyme E2 G2 | |
| G6PC | Glucose-6-Phosphatase Catalytic Subunit | |
| PRKAA1 | Protein Kinase AMP-Activated Catalytic Subunit Alpha 1 | |
| EGF | Epidermal Growth Factor | |
| CAV1 | Caveolin 1 | |
| RAB1B | RAB1B, Member RAS Oncogene Family | |
| BGLAP | Bone Gamma-Carboxyglutamate Protein | |
| CALM1 | Calmodulin 1 | |
| RAB1A | RAB1A, Member RAS Oncogene Family | |
| ATP2C1 | ATPase Secretory Pathway Ca2+ Transporting 1 | |
| EDEM3 | ER Degradation Enhancing Alpha-Mannosidase Like Protein 3 | |
| MAPK3 | Mitogen-Activated Protein Kinase 3 | |
| CRP | C-Reactive Protein | |
| ADAMTSL1 | ADAMTS Like 1 | |
| PLOD2 | Procollagen-Lysine,2-Oxoglutarate 5-Dioxygenase 2 | |
| CD40 | CD40 Molecule | |
| ENO2 | Enolase 2 |  |
| PIK3R1 | Phosphoinositide-3-Kinase Regulatory Subunit 1 | |
| SOAT1 | Sterol O-Acyltransferase 1 | |
| SREBF2 | Sterol Regulatory Element Binding Transcription Factor 2 | |
| GSK3B | Glycogen Synthase Kinase 3 Beta | |
| HSPD1 | Heat Shock Protein Family D (Hsp60) Member 1 | |
| MAP1LC3A | Microtubule Associated Protein 1 Light Chain 3 Alpha | |
| DDRGK1 | DDRGK Domain Containing 1 | |
| VAPA | VAMP Associated Protein A | |
| IFNG | Interferon Gamma | |
| COL1A1 | Collagen Type I Alpha 1 Chain | |
| SLN | Sarcolipin | |
| ALG13 | ALG13 UDP-N-Acetylglucosaminyltransferase Subunit | |
| CXCR4 | C-X-C Motif Chemokine Receptor 4 | |
| EDEM2 | ER Degradation Enhancing Alpha-Mannosidase Like Protein 2 | |
| CASP7 | Caspase 7 |  |
| TUSC3 | Tumor Suppressor Candidate 3 | |
| PNKD | PNKD Metallo-Beta-Lactamase Domain Containing | |
| IER3IP1 | Immediate Early Response 3 Interacting Protein 1 | |
| KCNQ1 | Potassium Voltage-Gated Channel Subfamily Q Member 1 | |
| LDLR | Low Density Lipoprotein Receptor | |
| FKBP14 | FKBP Prolyl Isomerase 14 | |
| AIFM1 | Apoptosis Inducing Factor Mitochondria Associated 1 | |
| IL10 | Interleukin 10 | |
| HIF1A | Hypoxia Inducible Factor 1 Subunit Alpha | |
| PRDX4 | Peroxiredoxin 4 | |
| ALG3 | ALG3 Alpha-1,3- Mannosyltransferase | |
| BMP2 | Bone Morphogenetic Protein 2 | |
| GAPDH | Glyceraldehyde-3-Phosphate Dehydrogenase | |
| ALG11 | ALG11 Alpha-1,2-Mannosyltransferase | |
| FGFR3 | Fibroblast Growth Factor Receptor 3 | |
| NCK1 | NCK Adaptor Protein 1 | |
| BCL2L11 | BCL2 Like 11 | |
| CDKN1A | Cyclin Dependent Kinase Inhibitor 1A | |
| CYBB | Cytochrome B-245 Beta Chain | |
| ERGIC3 | ERGIC And Golgi 3 | |
| HLA-A | Major Histocompatibility Complex, Class I, A | |
| FICD | FIC Domain Containing | |
| POR | Cytochrome P450 Oxidoreductase | |
| ATM | ATM Serine/Threonine Kinase | |
| APEX1 | Apurinic/Apyrimidinic Endodeoxyribonuclease 1 | |
| VCAM1 | Vascular Cell Adhesion Molecule 1 | |
| NQO1 | NAD(P)H Quinone Dehydrogenase 1 | |
| PTPN11 | Protein Tyrosine Phosphatase Non-Receptor Type 11 | |
| PCSK9 | Proprotein Convertase Subtilisin/Kexin Type 9 | |
| SLC37A4 | Solute Carrier Family 37 Member 4 | |
| TRAPPC2 | Trafficking Protein Particle Complex 2 | |
| MAP2K7 | Mitogen-Activated Protein Kinase Kinase 7 | |
| CPT2 | Carnitine Palmitoyltransferase 2 | |
| BOK | BCL2 Family Apoptosis Regulator BOK | |
| COL1A2 | Collagen Type I Alpha 2 Chain | |
| DNAJB12 | DnaJ Heat Shock Protein Family (Hsp40) Member B12 | |
| BECN1 | Beclin 1 |  |
| RECK | Reversion Inducing Cysteine Rich Protein With Kazal Motifs | |
| TOMM40 | Translocase Of Outer Mitochondrial Membrane 40 | |
| SRC | SRC Proto-Oncogene, Non-Receptor Tyrosine Kinase | |
| GPR37 | G Protein-Coupled Receptor 37 | |
| SERPINI1 | Serpin Family I Member 1 | |
| G6PD | Glucose-6-Phosphate Dehydrogenase | |
| COPB1 | COPI Coat Complex Subunit Beta 1 | |
| PKP2 | Plakophilin 2 | |
| PRL | Prolactin |  |
| SEC61G | SEC61 Translocon Subunit Gamma | |
| UBE2D2 | Ubiquitin Conjugating Enzyme E2 D2 | |
| ABCC8 | ATP Binding Cassette Subfamily C Member 8 | |
| ERLIN1 | ER Lipid Raft Associated 1 | |
| TEX264 | Testis Expressed 264, ER-Phagy Receptor | |
| STT3A | STT3 Oligosaccharyltransferase Complex Catalytic Subunit A | |
| DNM1L | Dynamin 1 Like | |
| NLRP3 | NLR Family Pyrin Domain Containing 3 | |
| UBE2D3 | Ubiquitin Conjugating Enzyme E2 D3 | |
| TMEM43 | Transmembrane Protein 43 | |
| RUNX2 | RUNX Family Transcription Factor 2 | |
| RHOA | Ras Homolog Family Member A | |
| UBE2J2 | Ubiquitin Conjugating Enzyme E2 J2 | |
| ARL6IP1 | ADP Ribosylation Factor Like GTPase 6 Interacting Protein 1 | |
| NUPR1 | Nuclear Protein 1, Transcriptional Regulator | |
| TXNIP | Thioredoxin Interacting Protein | |
| TNFSF11 | TNF Superfamily Member 11 | |
| PPARA | Peroxisome Proliferator Activated Receptor Alpha | |
| GABARAPL1 | GABA Type A Receptor Associated Protein Like 1 | |
| TRPM4 | Transient Receptor Potential Cation Channel Subfamily M Member 4 | |
| TRAM1 | Translocation Associated Membrane Protein 1 | |
| SRPRA | SRP Receptor Subunit Alpha | |
| LIPC | Lipase C, Hepatic Type | |
| STAU1 | Staufen Double-Stranded RNA Binding Protein 1 | |
| FKRP | Fukutin Related Protein | |
| TMEM208 | Transmembrane Protein 208 | |
| UFL1 | UFM1 Specific Ligase 1 | |
| STX17 | Syntaxin 17 | |
| TYR | Tyrosinase | |
| MAP1LC3B | Microtubule Associated Protein 1 Light Chain 3 Beta | |
| CLCN1 | Chloride Voltage-Gated Channel 1 | |
| CRHR1 | Corticotropin Releasing Hormone Receptor 1 | |
| RNF139 | Ring Finger Protein 139 | |
| TCF7L2 | Transcription Factor 7 Like 2 | |
| VKORC1 | Vitamin K Epoxide Reductase Complex Subunit 1 | |
| HSPA6 | Heat Shock Protein Family A (Hsp70) Member 6 | |
| CAPN2 | Calpain 2 |  |
| RB1 | RB Transcriptional Corepressor 1 | |
| GANAB | Glucosidase II Alpha Subunit | |
| NPPB | Natriuretic Peptide B | |
| DCN | Decorin |  |
| NR3C2 | Nuclear Receptor Subfamily 3 Group C Member 2 | |
| OSBP | Oxysterol Binding Protein | |
| UFD1 | Ubiquitin Recognition Factor In ER Associated Degradation 1 | |
| NOTCH1 | Notch Receptor 1 | |
| TMED10 | Transmembrane P24 Trafficking Protein 10 | |
| HLA-DRB3 | Major Histocompatibility Complex, Class II, DR Beta 3 | |
| IGF1 | Insulin Like Growth Factor 1 | |
| TAP2 | Transporter 2, ATP Binding Cassette Subfamily B Member | |
| TMED9 | Transmembrane P24 Trafficking Protein 9 | |
| FAS | Fas Cell Surface Death Receptor | |
| MDM2 | MDM2 Proto-Oncogene | |
| BSG | Basigin (Ok Blood Group) | |
| TMED4 | Transmembrane P24 Trafficking Protein 4 | |
| RNF5 | Ring Finger Protein 5 | |
| DHCR24 | 24-Dehydrocholesterol Reductase | |
| CCR6 | C-C Motif Chemokine Receptor 6 | |
| FOXO1 | Forkhead Box O1 | |
| USO1 | USO1 Vesicle Transport Factor | |
| AGR2 | Anterior Gradient 2, Protein Disulphide Isomerase Family Member | |
| ITIH4 | Inter-Alpha-Trypsin Inhibitor Heavy Chain 4 | |
| TRPC4 | Transient Receptor Potential Cation Channel Subfamily C Member 4 | |
| CD8A | CD8a Molecule | |
| LEP | Leptin |  |
| TIA1 | TIA1 Cytotoxic Granule Associated RNA Binding Protein | |
| SCAP | SREBF Chaperone | |
| ZFAND2B | Zinc Finger AN1-Type Containing 2B | |
| OSBPL8 | Oxysterol Binding Protein Like 8 | |
| TMED2 | Transmembrane P24 Trafficking Protein 2 | |
| MAN2A1 | Mannosidase Alpha Class 2A Member 1 | |
| UBQLN2 | Ubiquilin 2 | |
| NPLOC4 | NPL4 Homolog, Ubiquitin Recognition Factor | |
| RNF186 | Ring Finger Protein 186 | |
| KTN1 | Kinectin 1 |  |
| PRKAA2 | Protein Kinase AMP-Activated Catalytic Subunit Alpha 2 | |
| FABP1 | Fatty Acid Binding Protein 1 | |
| C9orf72 | C9orf72-SMCR8 Complex Subunit | |
| CP | Ceruloplasmin | |
| TMX1 | Thioredoxin Related Transmembrane Protein 1 | |
| ARSA | Arylsulfatase A | |
| CALHM1 | Calcium Homeostasis Modulator 1 | |
| CUL1 | Cullin 1 |  |
| G3BP1 | G3BP Stress Granule Assembly Factor 1 | |
| GCG | Glucagon |  |
| MATN3 | Matrilin 3 |  |
| TMEM214 | Transmembrane Protein 214 | |
| RAB6A | RAB6A, Member RAS Oncogene Family | |
| RAC1 | Rac Family Small GTPase 1 | |
| CCDC47 | Coiled-Coil Domain Containing 47 | |
| TMTC3 | Transmembrane O-Mannosyltransferase Targeting Cadherins 3 | |
| ACTC1 | Actin Alpha Cardiac Muscle 1 | |
| CASP2 | Caspase 2 |  |
| ORMDL3 | ORMDL Sphingolipid Biosynthesis Regulator 3 | |
| RNFT1 | Ring Finger Protein, Transmembrane 1 | |
| GPT | Glutamic--Pyruvic Transaminase | |
| PON2 | Paraoxonase 2 | |
| HMGB1 | High Mobility Group Box 1 | |
| MGAT5 | Alpha-1,6-Mannosylglycoprotein 6-Beta-N-Acetylglucosaminyltransferase | |
| SSR2 | Signal Sequence Receptor Subunit 2 | |
| JPH4 | Junctophilin 4 | |
| UFM1 | Ubiquitin Fold Modifier 1 | |
| PTGS1 | Prostaglandin-Endoperoxide Synthase 1 | |
| TRIB3 | Tribbles Pseudokinase 3 | |
| ASPH | Aspartate Beta-Hydroxylase | |
| MYOC | Myocilin |  |
| CREB1 | CAMP Responsive Element Binding Protein 1 | |
| PIK3CG | Phosphatidylinositol-4,5-Bisphosphate 3-Kinase Catalytic Subunit Gamma | |
| HTRA2 | HtrA Serine Peptidase 2 | |
| TFG | Trafficking From ER To Golgi Regulator | |
| ANXA5 | Annexin A5 | |
| GABARAP | GABA Type A Receptor-Associated Protein | |
| ARSG | Arylsulfatase G | |
| ITGB1 | Integrin Subunit Beta 1 | |
| MAN1A1 | Mannosidase Alpha Class 1A Member 1 | |
| PRKCD | Protein Kinase C Delta | |
| AGER | Advanced Glycosylation End-Product Specific Receptor | |
| CYP1A1 | Cytochrome P450 Family 1 Subfamily A Member 1 | |
| INSR | Insulin Receptor | |
| PPARGC1A | PPARG Coactivator 1 Alpha | |
| SCAMP5 | Secretory Carrier Membrane Protein 5 | |
| STAT3 | Signal Transducer And Activator Of Transcription 3 | |
| TTN | Titin |  |
| NPY | Neuropeptide Y | |
| TG | Thyroglobulin | |
| TMCO1 | Transmembrane And Coiled-Coil Domains 1 | |
| FOXO3 | Forkhead Box O3 | |
| DNAJA1 | DnaJ Heat Shock Protein Family (Hsp40) Member A1 | |
| PIEZO1 | Piezo Type Mechanosensitive Ion Channel Component 1 | |
| BAG1 | BAG Cochaperone 1 | |
| RPSA | Ribosomal Protein SA | |
| KNG1 | Kininogen 1 | |
| RYR3 | Ryanodine Receptor 3 | |
| TOR1B | Torsin Family 1 Member B | |
| SSR1 | Signal Sequence Receptor Subunit 1 | |
| ADRB2 | Adrenoceptor Beta 2 | |
| NOS1 | Nitric Oxide Synthase 1 | |
| IREB2 | Iron Responsive Element Binding Protein 2 | |
| P4HTM | Prolyl 4-Hydroxylase, Transmembrane | |
| AGR3 | Anterior Gradient 3, Protein Disulphide Isomerase Family Member | |
| CDIPT | CDP-Diacylglycerol--Inositol 3-Phosphatidyltransferase | |
| SRP68 | Signal Recognition Particle 68 | |
| CCDC88B | Coiled-Coil Domain Containing 88B | |
| CERT1 | Ceramide Transporter 1 | |
| LRP5 | LDL Receptor Related Protein 5 | |
| NGF | Nerve Growth Factor | |
| RAB10 | RAB10, Member RAS Oncogene Family | |
| TLR9 | Toll Like Receptor 9 | |
| SHH | Sonic Hedgehog Signaling Molecule | |
| MMP9 | Matrix Metallopeptidase 9 | |
| UGT1A1 | UDP Glucuronosyltransferase Family 1 Member A1 | |
| PINK1 | PTEN Induced Kinase 1 | |
| ERGIC2 | ERGIC And Golgi 2 | |
| SERPINA7 | Serpin Family A Member 7 | |
| YKT6 | YKT6 V-SNARE Homolog | |
| SEC22B | SEC22 Homolog B, Vesicle Trafficking Protein (Gene/Pseudogene) | |
| SGPP1 | Sphingosine-1-Phosphate Phosphatase 1 | |
| MGST1 | Microsomal Glutathione S-Transferase 1 | |
| RBX1 | Ring-Box 1 | |
| STARD3 | StAR Related Lipid Transfer Domain Containing 3 | |
| CD36 | CD36 Molecule | |
| ESYT2 | Extended Synaptotagmin 2 | |
| GBA | Glucosylceramidase Beta | |
| SCG5 | Secretogranin V | |
| KDR | Kinase Insert Domain Receptor | |
| SCD | Stearoyl-CoA Desaturase | |
| TERT | Telomerase Reverse Transcriptase | |
| PKD2 | Polycystin 2, Transient Receptor Potential Cation Channel | |
| CREBRF | CREB3 Regulatory Factor | |
| GSTM1 | Glutathione S-Transferase Mu 1 | |
| GPX1 | Glutathione Peroxidase 1 | |
| RHBDD1 | Rhomboid Domain Containing 1 | |
| TGM2 | Transglutaminase 2 | |
| UBE4B | Ubiquitination Factor E4B | |
| DSG2 | Desmoglein 2 | |
| STX18 | Syntaxin 18 | |
| COL9A1 | Collagen Type IX Alpha 1 Chain | |
| MARCHF6 | Membrane Associated Ring-CH-Type Finger 6 | |
| FKBP10 | FKBP Prolyl Isomerase 10 | |
| UBA52 | Ubiquitin A-52 Residue Ribosomal Protein Fusion Product 1 | |
| UBE2D1 | Ubiquitin Conjugating Enzyme E2 D1 | |
| WNT1 | Wnt Family Member 1 | |
| CDK1 | Cyclin Dependent Kinase 1 | |
| DES | Desmin |  |
| HSPA1L | Heat Shock Protein Family A (Hsp70) Member 1 Like | |
| ACAN | Aggrecan |  |
| ATXN3 | Ataxin 3 |  |
| ACE | Angiotensin I Converting Enzyme | |
| JPH3 | Junctophilin 3 | |
| UBQLN4 | Ubiquilin 4 | |
| ARSH | Arylsulfatase Family Member H | |
| FBXO6 | F-Box Protein 6 | |
| FUS | FUS RNA Binding Protein | |
| GUSB | Glucuronidase Beta | |
| PML | Promyelocytic Leukemia | |
| AGTR1 | Angiotensin II Receptor Type 1 | |
| SEC23IP | SEC23 Interacting Protein | |
| TTR | Transthyretin | |
| AUP1 | AUP1 Lipid Droplet Regulating VLDL Assembly Factor | |
| NGLY1 | N-Glycanase 1 | |
| PRKCSH | Protein Kinase C Substrate 80K-H | |
| IVL | Involucrin |  |
| UBXN4 | UBX Domain Protein 4 | |
| JKAMP | JNK1/MAPK8 Associated Membrane Protein | |
| HSPA2 | Heat Shock Protein Family A (Hsp70) Member 2 | |
| TNFSF10 | TNF Superfamily Member 10 | |
| GET4 | Guided Entry Of Tail-Anchored Proteins Factor 4 | |
| HSD17B10 | Hydroxysteroid 17-Beta Dehydrogenase 10 | |
| DAB2IP | DAB2 Interacting Protein | |
| UBL4A | Ubiquitin Like 4A | |
| BSCL2 | BSCL2 Lipid Droplet Biogenesis Associated, Seipin | |
| GSTP1 | Glutathione S-Transferase Pi 1 | |
| MFN2 | Mitofusin 2 | |
| UBE2K | Ubiquitin Conjugating Enzyme E2 K | |
| CAMK2G | Calcium/Calmodulin Dependent Protein Kinase II Gamma | |
| TMEM117 | Transmembrane Protein 117 | |
| GOSR1 | Golgi SNAP Receptor Complex Member 1 | |
| OPA3 | Outer Mitochondrial Membrane Lipid Metabolism Regulator OPA3 | |
| SCN4A | Sodium Voltage-Gated Channel Alpha Subunit 4 | |
| GOLGA2 | Golgin A2 | |
| MYC | MYC Proto-Oncogene, BHLH Transcription Factor | |
| CYB5R3 | Cytochrome B5 Reductase 3 | |
| SVIP | Small VCP Interacting Protein | |
| NLRP1 | NLR Family Pyrin Domain Containing 1 | |
| ZDHHC6 | Zinc Finger DHHC-Type Palmitoyltransferase 6 | |
| EEF2 | Eukaryotic Translation Elongation Factor 2 | |
| TMEM259 | Transmembrane Protein 259 | |
| TNFRSF1A | TNF Receptor Superfamily Member 1A | |
| CDK5 | Cyclin Dependent Kinase 5 | |
| DSPP | Dentin Sialophosphoprotein | |
| EEF1A1 | Eukaryotic Translation Elongation Factor 1 Alpha 1 | |
| NSFL1C | NSFL1 Cofactor | |
| F8 | Coagulation Factor VIII | |
| TMX3 | Thioredoxin Related Transmembrane Protein 3 | |
| OXT | Oxytocin/Neurophysin I Prepropeptide | |
| SLC2A1 | Solute Carrier Family 2 Member 1 | |
| EIF2AK1 | Eukaryotic Translation Initiation Factor 2 Alpha Kinase 1 | |
| ICAM1 | Intercellular Adhesion Molecule 1 | |
| CACNB4 | Calcium Voltage-Gated Channel Auxiliary Subunit Beta 4 | |
| CDKAL1 | CDK5 Regulatory Subunit Associated Protein 1 Like 1 | |
| FMR1 | FMRP Translational Regulator 1 | |
| FURIN | Furin, Paired Basic Amino Acid Cleaving Enzyme | |
| PLA2G6 | Phospholipase A2 Group VI | |
| COPA | COPI Coat Complex Subunit Alpha | |
| PTGIS | Prostaglandin I2 Synthase | |
| MAPKAPK2 | MAPK Activated Protein Kinase 2 | |
| SSR3 | Signal Sequence Receptor Subunit 3 | |
| TFEB | Transcription Factor EB | |
| CYP1A2 | Cytochrome P450 Family 1 Subfamily A Member 2 | |
| DUSP19 | Dual Specificity Phosphatase 19 | |
| DNAJB2 | DnaJ Heat Shock Protein Family (Hsp40) Member B2 | |
| HLA-DRA | Major Histocompatibility Complex, Class II, DR Alpha | |
| HSPH1 | Heat Shock Protein Family H (Hsp110) Member 1 | |
| TMCC1 | Transmembrane And Coiled-Coil Domain Family 1 | |
| PSMD2 | Proteasome 26S Subunit, Non-ATPase 2 | |
| DHCR7 | 7-Dehydrocholesterol Reductase | |
| DPM2 | Dolichyl-Phosphate Mannosyltransferase Subunit 2, Regulatory | |
| TGFB3 | Transforming Growth Factor Beta 3 | |
| APAF1 | Apoptotic Peptidase Activating Factor 1 | |
| CALR3 | Calreticulin 3 | |
| COL9A2 | Collagen Type IX Alpha 2 Chain | |
| MYH6 | Myosin Heavy Chain 6 | |
| CAPN1 | Calpain 1 |  |
| PRDX1 | Peroxiredoxin 1 | |
| GORASP2 | Golgi Reassembly Stacking Protein 2 | |
| NOTCH3 | Notch Receptor 3 | |
| SEC31B | SEC31 Homolog B, COPII Coat Complex Component | |
| AQP11 | Aquaporin 11 | |
| ICMT | Isoprenylcysteine Carboxyl Methyltransferase | |
| MCL1 | MCL1 Apoptosis Regulator, BCL2 Family Member | |
| SKP1 | S-Phase Kinase Associated Protein 1 | |
| SOX9 | SRY-Box Transcription Factor 9 | |
| FLNB | Filamin B |  |
| JPH1 | Junctophilin 1 | |
| EGR1 | Early Growth Response 1 | |
| SDF2L1 | Stromal Cell Derived Factor 2 Like 1 | |
| ECPAS | Ecm29 Proteasome Adaptor And Scaffold | |
| CCND1 | Cyclin D1 |  |
| CDH2 | Cadherin 2 | |
| FASLG | Fas Ligand | |
| YOD1 | YOD1 Deubiquitinase | |
| MAP2K1 | Mitogen-Activated Protein Kinase Kinase 1 | |
| PRKCA | Protein Kinase C Alpha | |
| ALOX15 | Arachidonate 15-Lipoxygenase | |
| EPO | Erythropoietin | |
| SGTA | Small Glutamine Rich Tetratricopeptide Repeat Containing Alpha | |
| TLR2 | Toll Like Receptor 2 | |
| GOLPH3 | Golgi Phosphoprotein 3 | |
| RSAD2 | Radical S-Adenosyl Methionine Domain Containing 2 | |
| GP6 | Glycoprotein VI Platelet | |
| TTPA | Alpha Tocopherol Transfer Protein | |
| CPQ | Carboxypeptidase Q | |
| KPNB1 | Karyopherin Subunit Beta 1 | |
| EIF4E | Eukaryotic Translation Initiation Factor 4E | |
| PPP1CA | Protein Phosphatase 1 Catalytic Subunit Alpha | |
| FOXRED2 | FAD Dependent Oxidoreductase Domain Containing 2 | |
| KRT14 | Keratin 14 | |
| UBXN8 | UBX Domain Protein 8 | |
| CTSD | Cathepsin D | |
| HM13 | Histocompatibility Minor 13 | |
| PKD1 | Polycystin 1, Transient Receptor Potential Channel Interacting | |
| DSG3 | Desmoglein 3 | |
| COL9A3 | Collagen Type IX Alpha 3 Chain | |
| PRDM10 | PR/SET Domain 10 | |
| APOA4 | Apolipoprotein A4 | |
| FKBP5 | FKBP Prolyl Isomerase 5 | |
| MATN1 | Matrilin 1 |  |
| PTH | Parathyroid Hormone | |
| ELAVL1 | ELAV Like RNA Binding Protein 1 | |
| M6PR | Mannose-6-Phosphate Receptor, Cation Dependent | |
| SERPINA2 | Serpin Family A Member 2 (Gene/Pseudogene) | |
| AIF1 | Allograft Inflammatory Factor 1 | |
| MT-TK | Mitochondrially Encoded TRNA-Lys (AAA/G) | |
| SFTPC | Surfactant Protein C | |
| TPP1 | Tripeptidyl Peptidase 1 | |
| USP19 | Ubiquitin Specific Peptidase 19 | |
| ATXN2 | Ataxin 2 |  |
| CDC42 | Cell Division Cycle 42 | |
| GPER1 | G Protein-Coupled Estrogen Receptor 1 | |
| PRDX5 | Peroxiredoxin 5 | |
| U2AF1 | U2 Small Nuclear RNA Auxiliary Factor 1 | |
| PXN | Paxillin |  |
| SHISA5 | Shisa Family Member 5 | |
| BRCA1 | BRCA1 DNA Repair Associated | |
| SHC1 | SHC Adaptor Protein 1 | |
| HEXA | Hexosaminidase Subunit Alpha | |
| OSBPL3 | Oxysterol Binding Protein Like 3 | |
| COL10A1 | Collagen Type X Alpha 1 Chain | |
| QDPR | Quinoid Dihydropteridine Reductase | |
| SET | SET Nuclear Proto-Oncogene | |
| BET1 | Bet1 Golgi Vesicular Membrane Trafficking Protein | |
| CES1 | Carboxylesterase 1 | |
| LPIN1 | Lipin 1 |  |
| RAB2A | RAB2A, Member RAS Oncogene Family | |
| SCFD1 | Sec1 Family Domain Containing 1 | |
| EMD | Emerin |  |
| NPC1 | NPC Intracellular Cholesterol Transporter 1 | |
| STBD1 | Starch Binding Domain 1 | |
| UBE2G1 | Ubiquitin Conjugating Enzyme E2 G1 | |
| CYB5A | Cytochrome B5 Type A | |
| MAPK8IP1 | Mitogen-Activated Protein Kinase 8 Interacting Protein 1 | |
| SELENOF | Selenoprotein F | |
| CYB5R4 | Cytochrome B5 Reductase 4 | |
| DMPK | DM1 Protein Kinase | |
| SPAST | Spastin |  |
| CTSK | Cathepsin K | |
| G6PC3 | Glucose-6-Phosphatase Catalytic Subunit 3 | |
| VDAC1 | Voltage Dependent Anion Channel 1 | |
| FPR2 | Formyl Peptide Receptor 2 | |
| SERPINE1 | Serpin Family E Member 1 | |
| COG7 | Component Of Oligomeric Golgi Complex 7 | |
| SGPP2 | Sphingosine-1-Phosphate Phosphatase 2 | |
| CAV3 | Caveolin 3 | |
| DPAGT1 | Dolichyl-Phosphate N-Acetylglucosaminephosphotransferase 1 | |
| RCN1 | Reticulocalbin 1 | |
| CTSB | Cathepsin B | |
| NCK2 | NCK Adaptor Protein 2 | |
| PTK2 | Protein Tyrosine Kinase 2 | |
| SEC11A | SEC11 Homolog A, Signal Peptidase Complex Subunit | |
| SRPRB | SRP Receptor Subunit Beta | |
| PSMA5 | Proteasome 20S Subunit Alpha 5 | |
| STIP1 | Stress Induced Phosphoprotein 1 | |
| CLGN | Calmegin |  |
| PTEN | Phosphatase And Tensin Homolog | |
| ZC3H12A | Zinc Finger CCCH-Type Containing 12A | |
| HSD17B12 | Hydroxysteroid 17-Beta Dehydrogenase 12 | |
| RCN2 | Reticulocalbin 2 | |
| AFP | Alpha Fetoprotein | |
| UBAC2 | UBA Domain Containing 2 | |
| FN1 | Fibronectin 1 | |
| RELA | RELA Proto-Oncogene, NF-KB Subunit | |
| MMP2 | Matrix Metallopeptidase 2 | |
| PIGN | Phosphatidylinositol Glycan Anchor Biosynthesis Class N | |
| TMED7 | Transmembrane P24 Trafficking Protein 7 | |
