# Supplementary material for: Prognostic Risk Signature and Comprehensive Analyses of Endoplasmic Reticulum Stress-Related Genes in Lung Adenocarcinoma
Source: J Immunol Res. 2022 May 4;2022:6567916. doi: 10.1155/2022/6567916 (PMC9096573; doi:10.1155/2022/6567916)
Supplement: Supplementary 6 — Table S2: drugs. [file 6567916.f6.docx]

**Table S2 The information of Drugs.**

| Gene | Drug | cor | pvalue |
| --- | --- | --- | --- |
| AGER | Nelarabine | 0.675973 | 3.10E-09 |
| SFTPC | Vemurafenib | 0.602409 | 3.54E-07 |
| AGER | Dexamethasone Decadron | 0.583443 | 9.96E-07 |
| SFTPC | Dabrafenib | 0.520943 | 1.98E-05 |
| SFTPC | Denileukin Diftitox Ontak | 0.520537 | 2.01E-05 |
| AGER | Fludarabine | 0.516428 | 2.40E-05 |
| SSR4 | Carmustine | 0.402107 | 0.001449 |
| GJB2 | tfdu | -0.39377 | 0.001853 |
| AGER | Cladribine | 0.390045 | 0.002065 |
| ADRB2 | Homoharringtonine | -0.38965 | 0.002089 |
| ADRB2 | Oxaliplatin | -0.38668 | 0.002274 |
| AGER | Vorinostat | 0.386515 | 0.002285 |
| ADRB2 | Mithramycin | -0.38205 | 0.002593 |
| SFTPC | Encorafenib | 0.381533 | 0.002631 |
| GJB2 | Etoposide | -0.37166 | 0.003457 |
| SFTPC | umbralisib | -0.36937 | 0.003679 |
| SLC6A4 | Imiquimod | 0.366009 | 0.004028 |
| ADRB2 | Actinomycin D | -0.36484 | 0.004156 |
| ADRB2 | Fluorouracil | -0.36406 | 0.004243 |
| SSR4 | Estramustine | 0.363943 | 0.004256 |
| SSR4 | Carboplatin | 0.361122 | 0.004586 |
| SFTPC | Selumetinib | 0.36005 | 0.004718 |
| AGER | Fluphenazine | 0.357075 | 0.005099 |
| SSR4 | TESTOLACTONE | 0.356392 | 0.005191 |
| ADRB2 | Tamoxifen | -0.3562 | 0.005217 |
| ADRB2 | Allopurinol | 0.354009 | 0.005521 |
| SSR4 | Nelfinavir | 0.353033 | 0.005662 |
| AGER | 6-THIOGUANINE | 0.351542 | 0.005883 |
| AGER | Asparaginase | 0.343211 | 0.007261 |
| SLC2A1 | Denileukin Diftitox Ontak | -0.34115 | 0.007642 |
| AGER | Mithramycin | -0.3404 | 0.007785 |
| SSR4 | Hydroxyurea | 0.339578 | 0.007945 |
| SSR4 | Lomustine | 0.339555 | 0.007949 |
| GJB2 | Triethylenemelamine | -0.33832 | 0.008194 |
| GJB2 | Uracil mustard | -0.3373 | 0.008402 |
| SSR4 | Chlorambucil | 0.337246 | 0.008413 |
| SSR4 | Cyclophosphamide | 0.33696 | 0.008472 |
| AGER | Cytarabine | 0.336882 | 0.008488 |
| ADRB2 | Acrichine | -0.33629 | 0.008611 |
| GJB2 | Gemcitabine | -0.33567 | 0.008744 |
| GJB2 | Fludarabine | -0.33546 | 0.008787 |
| SSR4 | Calusterone | 0.334803 | 0.008929 |
| ADRB2 | Vinblastine | -0.33433 | 0.009032 |
| GJB2 | Melphalan | -0.32995 | 0.010036 |
| SLC6A4 | Dexrazoxane | 0.32969 | 0.010099 |
| SFTPC | ARRY-162 | 0.328487 | 0.010393 |
| SSR4 | Megestrol acetate | 0.326421 | 0.010915 |
| GJB2 | Thiotepa | -0.32608 | 0.011002 |
| SSR4 | Pipobroman | 0.322421 | 0.011991 |
| GJB2 | Cisplatin | -0.32137 | 0.012288 |
| SSR4 | Fluphenazine | 0.321195 | 0.012338 |
| SFTPC | Dasatinib | -0.31944 | 0.01285 |
| ADRB2 | Dasatinib | 0.316614 | 0.013714 |
| AGER | Depsipeptide | -0.31657 | 0.013727 |
| AGER | Hydroxyurea | 0.313756 | 0.014638 |
| SSR4 | Uracil mustard | 0.310866 | 0.015626 |
| SSR4 | Dromostanolone Propionate | 0.309551 | 0.016094 |
| ADRB2 | Tegafur | -0.30891 | 0.016326 |
| AGER | Clofarabine | 0.308026 | 0.016652 |
| ADRB2 | Epirubicin | -0.30801 | 0.016658 |
| GJB2 | DACARBAZINE | -0.30737 | 0.016898 |
| SFTPC | Cobimetinib (isomer 1) | 0.306735 | 0.017137 |
| GJB2 | Chlorambucil | -0.30595 | 0.017439 |
| ADRB2 | Vinorelbine | -0.30576 | 0.017511 |
| AGER | Chlorambucil | 0.305578 | 0.017582 |
| SSR4 | Melphalan | 0.303005 | 0.018607 |
| ADRB2 | Vemurafenib | -0.30151 | 0.019224 |
| GJB2 | Cytarabine | -0.29844 | 0.020551 |
| SLC6A4 | DECITABINE | 0.298115 | 0.020696 |
| SSR4 | Isotretinoin | 0.29429 | 0.022465 |
| ADRB2 | Bortezomib | -0.29374 | 0.02273 |
| SFTPC | brigatinib | -0.29311 | 0.023038 |
| GJB2 | Cladribine | -0.29274 | 0.023216 |
| CDKN3 | LEE-011 | 0.291195 | 0.023989 |
| ADRB2 | Simvastatin | 0.289999 | 0.024601 |
| GJB2 | Topotecan | -0.28825 | 0.025521 |
| SSR4 | Sulfatinib | 0.287234 | 0.026066 |
| SLC2A1 | Simvastatin | 0.286889 | 0.026254 |
| ADRB2 | Fludarabine | 0.286659 | 0.02638 |
| SLC6A4 | Isotretinoin | 0.284047 | 0.027845 |
| CDKN3 | Denileukin Diftitox Ontak | -0.28393 | 0.027911 |
| SLC2A1 | DIGOXIN | -0.28291 | 0.028506 |
| AGER | Gemcitabine | 0.279082 | 0.03082 |
| SSR4 | Axitinib | 0.278683 | 0.03107 |
| ADRB2 | Ixabepilone | -0.27765 | 0.031723 |
| SSR4 | Vismodegib | 0.276292 | 0.032605 |
| SSR4 | Triethylenemelamine | 0.274221 | 0.033985 |
| ADRB2 | Pipamperone | -0.27394 | 0.034177 |
| GJB2 | Teniposide | -0.27393 | 0.034186 |
| CDKN3 | Temsirolimus | -0.27192 | 0.035572 |
| AGER | Actinomycin D | -0.27077 | 0.036393 |
| AGER | Abiraterone | -0.26794 | 0.038469 |
| SLC2A1 | Irofulven | 0.265897 | 0.040031 |
| GJB2 | 7-Ethyl-10-hydroxycamptothecin | -0.26458 | 0.041066 |
| ADRB2 | Dabrafenib | -0.26449 | 0.041133 |
| SSR4 | Neratinib | -0.26436 | 0.041238 |
| AGER | Uracil mustard | 0.264027 | 0.041505 |
| ADRB2 | Erlotinib | 0.263303 | 0.042087 |
| ADRB2 | Dacomitinib | 0.261477 | 0.043587 |
| SLC2A1 | Arsenic trioxide | -0.26054 | 0.044373 |
| SLC2A1 | Bendamustine | -0.26039 | 0.044497 |
| GJB2 | DIGOXIN | -0.2601 | 0.044746 |
| SLC2A1 | IPI-145 | 0.259448 | 0.045304 |
| GJB2 | Idarubicin | -0.25835 | 0.046252 |
| GJB2 | Irinotecan | -0.25785 | 0.046694 |
| SLC2A1 | Bortezomib | -0.25777 | 0.046764 |
| SLC2A1 | Ixazomib citrate | -0.25756 | 0.046953 |
| SLC2A1 | Dasatinib | 0.25747 | 0.047031 |
| ADRB2 | Nilotinib | -0.25652 | 0.047879 |
| GJB2 | Hydroxyurea | -0.25638 | 0.048001 |
| SFTPC | Acetalax | -0.25636 | 0.048022 |
| GJB2 | Temsirolimus | -0.25618 | 0.048182 |
| AGER | Arsenic trioxide | 0.256127 | 0.048234 |
| SLC6A4 | Fluphenazine | 0.255949 | 0.048395 |
| SSR4 | Arsenic trioxide | 0.255259 | 0.049025 |
