# Supplementary material for: Prognostic Risk Signature and Comprehensive Analyses of Endoplasmic Reticulum Stress-Related Genes in Lung Adenocarcinoma
Source: J Immunol Res. 2022 May 4;2022:6567916. doi: 10.1155/2022/6567916 (PMC9096573; doi:10.1155/2022/6567916)
Supplement: Supplementary 7 — Table S3: gene primer sequence. [file 6567916.f7.docx]

Table S3 The primer sequences.

| Gene | Primer Sequences |
| --- | --- |
| SSR4-Forward | GATGTGGGGCGTTATCAGGTGTC |
| SSR4-Reverse | CAGAGGCGGGATGATGGAAATGTC |
| SLC6A4-Forward | TGTCATCTTCACAGTGCTCGGTTAC |
| SLC6A4-Reverse | TGGCATGTTGGCTATCGCTTCTG |
| SLC2A1-Forward | GATGAAGGAAGAGAGTCGGCAGATG |
| SLC2A1-Reverse | CAGCACCACAGCGATGAGGATG |
| SFTPC-Forward | CACACGGAGATGGTTCTGGAGATG |
| SFTPC-Reverse | GATGGAGAAGGTGGCAGTGGTAAC |
| GJB2-Forward | GGTGGACCTACACAAGCAGCATC |
| GJB2-Reverse | GGAGAAGCCGTCGTACATGACATAG |
| CDKN3-Forward | ACTGCTATGGAGGACTTGGGAGATC |
| CDKN3-Reverse | TGGCTTGCTCTGGTGATATTGTGTC |
| AGER-Forward | TACCGAGTCCGTGTCTACCAGATTC |
| AGER-Reverse | CCATCCAAGTGCCAGCTAAGAGTC |
| ADRB2-Forward | CTATGCCAATGAGACCTGCTGTGAC |
| ADRB2-Reverse | AAAGACCCTGGAGTAGACGAAGACC |
| GAPDH-Forward | GCACCGTCAAGGCTGAGAAC |
| GAPDH-Reverse | TGGTGAAGACGCCAGTGGA |
