# Supplementary material for: Prognostic Risk Signature and Comprehensive Analyses of Endoplasmic Reticulum Stress-Related Genes in Lung Adenocarcinoma
Source: J Immunol Res. 2022 May 4;2022:6567916. doi: 10.1155/2022/6567916 (PMC9096573; doi:10.1155/2022/6567916)
Supplement: Supplementary 8 — Table S4: clinical information of LUAD patients. [file 6567916.f8.docx]

Table S4 Clinical information of LUAD patients.

| Patient ID | Gender | Age | Tumor tissue | Para-tumor tissue |
| --- | --- | --- | --- | --- |
| 1 | Male | 62 | Yes | Yes |
| 2 | Female | 58 | Yes | Yes |
| 3 | Male | 35 | Yes | Yes |
| 4 | Female | 67 | Yes | Yes |
| 5 | Female | 66 | Yes | Yes |
| 6 | Female | 59 | Yes | Yes |
| 7 | Male | 67 | Yes | Yes |
| 8 | Female | 65 | Yes | Yes |
| 9 | Male | 73 | Yes | Yes |
| 10 | Female | 68 | Yes | Yes |
| 11 | Male | 42 | Yes | Yes |
| 12 | Male | 58 | Yes | Yes |
